# NANUQ: a method for inferring species networks from gene trees under the coalescent model

**DOI:** 10.1186/s13015-019-0159-2

**Published:** 2019-12-06

**Authors:** Elizabeth S. Allman, Hector Baños, John A. Rhodes

**Affiliations:** Department of of Mathematics and Statistics, University of Alaska Fairbanks, 1792 Ambler Lane, Fairbanks, AK 99775 USA

**Keywords:** Hybridization, Network multispecies coalescent, Species network inference, Gene tree, Quartets, Level-1 network, NANUQ, Primary 92B10, 92D15

## Abstract

Species networks generalize the notion of species trees to allow for hybridization or other lateral gene transfer. Under the network multispecies coalescent model, individual gene trees arising from a network can have any topology, but arise with frequencies dependent on the network structure and numerical parameters. We propose a new algorithm for statistical inference of a level-1 species network under this model, from data consisting of gene tree topologies, and provide the theoretical justification for it. The algorithm is based on an analysis of quartets displayed on gene trees, combining several statistical hypothesis tests with combinatorial ideas such as a quartet-based intertaxon distance appropriate to networks, the NeighborNet algorithm for circular split systems, and the Circular Network algorithm for constructing a splits graph.

## Background

In this paper we provide the theory supporting a new, statistically consistent method of inferring most topological features of a level-1 hybridization network under the network multispecies coalescent (NMSC) model. The method uses as data a collection of unrooted topological gene trees, which may themselves have been inferred from sequences.

Unlike pseudo-likelihood methods [1, 2], our method does not require an assumed limit on the number of hybridization events in the network, nor does it involve a time-intensive search over the space of possible networks. Instead, it computes a certain distance between taxa which, under ideal circumstances, corresponds to a circular split system. When the expected distance is processed through particular algorithms to produce a splits graph, interpretation rules allow one to read off network information. The total theoretical running time of the algorithm is $\mathcal {O}(n^4m)$ for an input of *m* binary gene trees on *n* taxa, making it computationally feasible when *n* has moderate size.

While we illustrate the method’s utility through several examples with simulated and empirical data, our focus in this work is on providing its theoretical basis. This draws on a number of independent research works, but also requires new results on the nature of the splits graphs that are produced under ideal circumstances.

We call this new method the $\text{Network}$ inference $\text{Algorithm}$ via $\text{NeighbourNet}$ Using ${\text{Quartet}}$ distance, or by the acronym NANUQ.^1^ It involves the following steps, applied to a collection of unrooted gene tree topologies assumed to have arisen under the NMSC on an unknown binary level-1 network:

- For each subset of 4 taxa, determine the empirical quartet counts from the gene trees, which will reflect possible cycles on the network, as shown in [1, 3].
- Apply a statistical hypothesis test to these counts, as in [4], to judge evidence as to whether the quartet species network displays a 4-cycle.
- Use the test results on quartets to construct a network quartet distance between taxa, extending the ideas of [5].
- Apply the NeighborNet [6] and Circular Network algorithms [7] to construct a splits graph from the quartet distance.
- Interpret the abstract network produced in the previous step by certain rules developed in this paper to infer most topological features of the unknown network.

All steps but the last have been fully automated; in R for the steps (a–c), and SplitsTree4 [8] for step (d). While it is conceivable the last step could be as well, there are advantages to not doing so until more experience with the method has accumulated. For instance, some data sets may not support a hypothesis of evolution on a level-1 hybridization network, and a human interpretation of both the hypothesis test results of step (b) and the SplitsTree4 output of step (e) may suggest this. Simply returning a hybridization network most in accord with the output might be misleading if poor model fit is ignored.

NANUQ offers several important advantages over other network inference methods we know of. In particular, it can indicate poor model fit to the level-1 NMSC and, in the case of reasonable fit, indicate the number of hybridization events without conducting a time-consuming search. In contrast, pseudo-likelihood methods, which can be used for network inference [1, 2], are known broadly to be poor for judging model fit, though often perform well for inference. However, NANUQ only gives information on network topology, whereas pseudo-likelihood can be used to obtain metric information as well. We thus view NANUQ as complementary to existing approaches.

Several recent works [9, 10] have taken a Bayesian approach to inference of species networks from genetic sequence data, to obtain a joint posterior on both species networks and gene trees. As attractive as one might find this as a conceptual approach, it produces a formidable computational challenge for data sets with many taxa or gene trees. Indeed, the largest analyses in these works are quite small, involving only 7 taxa and 106 gene trees from a yeast data set which we also analyze. The alternative approaches offered by NANUQ and the pseudo-likelihood algorithms easily handle much larger data sets, with thousands of genes, as have already been assembled by researchers.

We note that NANUQ’s use of a splits graph is the first instance, to our knowledge, of such a graph being given a firm model-based interpretation as supporting a biological process underlying a data set. Splits graphs are generally viewed as exploratory devices for judging the extent to which a data set is “tree-like,” and authors often warn against interpreting them as supporting any particular biological mechanism [11]. We fully agree with this general statement; only in the framework of our multi-step algorithm do we claim that an interpretation of support for a hybridization network is justified by theory. While an earlier step in this direction was taken by [12], that work assumed no coalescent process modeling incomplete lineage sorting (ILS) was involved in the formation of gene trees, and provided a less detailed description of the form of a splits graph than is given here.

The theory we present is based on consideration of the quartets displayed on a collection of gene trees arising under the NMSC, but it differs in important ways from the more purely combinatorial work, such as [13], on undirected networks of level-1 and higher. First, we crucially focus on unrooted phylogenetic networks in the sense of [1, 3], which retain the direction of hybrid edges from the rooted species network underlying the biological model, rather than fully undirected networks of [13]. This leads to a different notion of the trees and quartets displayed on a network, and of the set of splits we associate to a network. Second, unlike most purely combinatorial studies, our algorithm takes into account that due to the coalescent process some gene trees will display quartets inconsistent with the species network. NANUQ provides a means of determining, up to statistical inference error, which quartets are displayed on the network. Third, if these quartets are known exactly, we are able to recover not only the undirected version of the network (modulo contraction of 2- and 3-cycles) but also directions of hybrid edges in cycles of size 5 or larger.

This paper proceeds as follows: We first outline and develop theory behind the NANUQ algorithm in a purely theoretical setting. This constitutes the majority of the work. We then more carefully outline the algorithm for data analysis, and conclude with a few examples of network inference.

In more detail, the theoretical portion of this work first formally defines the type of phylogenetic networks which underly our model, as well as unrooted semidirected networks induced from them. While this precise notion of unrooted network appeared in [3], it is not standard to the literature, yet it is essential to our work. Briefly recalling the network multispecies coalescent model (NMSC) and the notion of a quartet concordance factor (CF), we summarize results of [1, 3] indicating how these concordance factors reflect quartet network topology, and provide a new analysis indicating the extent to which one can avoid the one important case of ambiguity in interpreting CFs. After reviewing terminology for split systems, we then define a split system associated to an unrooted semidirected level-1 network. This is used to define a new quartet intertaxon distance for a level-1 topological network, which can be computed from quartet information alone. We then investigate the splits graph computed from the quartet distance of a binary level-1 network. This requires establishing some new theoretical results which enable us to directly relate the form of a level-1 hybridization network to the form of the splits graph found from its network quartet distance.

Finally, we present our algorithm in full, making use of all the theory above, as well as hypothesis testing using CFs as developed in [4], and the NeighborNet [6] and Circular Network [7] algorithms as implemented in SplitsTree4 [8]. We give a running time analysis for NANUQ and establish its statistical consistency. As our primary goal in this paper is to provide the theoretical background to our algorithm, we conclude with a minimal set of example analyses, using both simulated and biological data. A later work, directed at empiricists, will focus further on NANUQ’s performance in data analysis.

## Phylogenetic networks

### Rooted and unrooted phylogenetic networks

We begin by establishing terminology for phylogenetic networks. Throughout, $X=\{x_1,x_2,\dots ,x_n\}$ denotes a fixed set of taxa.

Our focus is on an explicit network [11], that can be interpreted as providing an evolutionary history of species relationships, including hybridization or other forms of lateral gene transfer that occur at discrete moments in time.

#### **Definition 1**

([3, 14]) A *topological binary rooted phylogenetic network* $N^+$ on taxon set *X* is a connected directed acyclic graph with vertices *V* and edges *E*, where *V* is the disjoint union $V = \{r\} \sqcup V_L \sqcup V_H \sqcup V_T$ and *E* is the disjoint union $E = E_H \sqcup E_T$, together with a bijective leaf-labeling function $f: V_L \rightarrow X$ with the following characteristics:

1. The *root* *r* has indegree 0 and outdegree 2.
2. A *leaf* $v \in V_L$ has indegree 1 and outdegree 0.
3. A *tree node* $v\in V_T$ has indegree 1 and outdegree 2.
4. A *hybrid node* $v\in V_H$ has indegree 2 and outdegree 1.
5. A *hybrid edge* $e \in E_H$ is an edge whose child is a hybrid node.
6. A *tree edge* $e \in E_T$ is an edge whose child is a tree node or a leaf.

#### **Definition 2**

Let $N^+$ be a topological binary rooted phylogenetic network. A *metric for* $N^+$ is a pair $(\lambda , \gamma )$, where $\lambda:E\rightarrow \mathbb {R}^{\ge 0}$ assigns *edge lengths* and $\gamma:E_H\rightarrow (0,1)$ assigns *hybridization parameters* satisfying

1. $$\lambda (e) >0$$ for $$e\in E_T$$,
2. $\gamma (e_1)+\gamma (e_2)=1$ whenever $e_1,e_2\in E_H$ have the same hybrid-node child.

If $(\lambda , \gamma )$ is a metric for $N^+$, then we refer to $(N^+,(\lambda , \gamma ))$ as a *metric binary rooted phylogenetic network*.

While the idea of unrooting a tree is simple, unrooting a network is more subtle. For example, it may not be clear how to proceed when the two edges incident to the root have the same child. We follow [3] in elucidating this concept.

In a directed network, we say that a node *v* is *above* a node *u*, and *u* is *below* *v*, if there exists a non-empty directed path in $N^+$ from *v* to *u*. We also say that an edge with parent node *x* and child *y* is *above* (*below*) a node *v* if *y* is above or equal to *v* (*x* is below or equal to *v*).

#### **Definition 3**

([14]) Let $N^+$ be a (metric or topological) binary rooted phylogenetic network on *X* and $Z\subseteq X$. Let *D* be the set of nodes which lie on every directed path from the root *r* of $N^+$ to any $z\in Z$. Then the *lowest stable ancestor of* *Z*  *on* $N^+$, denoted ${\text{LSA}}(Z)$, is the unique node $v\in D$ such that *v* is below all $u\in D$, $u\ne v$.

The lowest stable ancestor is a generalization (though not the only one) on a network of the concept of most recent common ancestor on a tree.

If *z* is a degree two node on a semidirected graph, with nodes *x* and *y* adjacent to *z*, then by *suppressing* *z* we mean deleting *z* and its incident edges, and introducing a new edge joining *x* and *y*. If the deleted edges formed a semidirected path, we direct this new edge consistently with that path; otherwise the new edge is undirected.

#### **Definition 4**

Let $N^+$ be a binary topological rooted phylogenetic network on a set of taxa *X*. Then $N^-$, the *topological unrooted phylogenetic network induced from* $N^+$, is the semidirected network obtained by

1. Deleting all edges and nodes above ${\text{LSA}}(X)$,
2. Undirecting all tree edges, and
3. Suppressing $${\text{LSA}}(X)$$.

If $N^+$ has a metric structure, then $N^-$ inherits one in an obvious way. Edge lengths on $N^-$ are the sum of conjoined edge lengths in $N^+$, and hybridization parameters are the same as those on $N^+$.

**Fig. 1:**
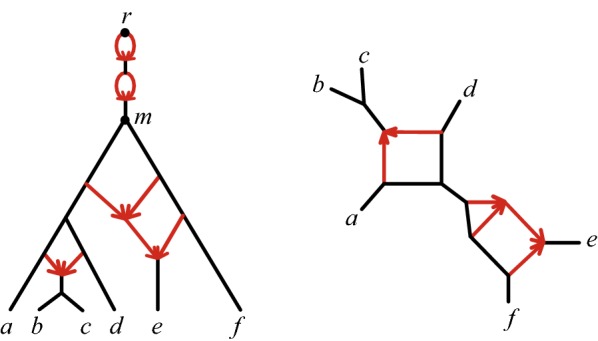
(L) A rooted phylogenetic network $N^+$ with root *r* and lowest stable ancestor *m*, and (R) the unrooted network $N^-$ induced from $N^+$

Note that in some other phylogenetic works the term “unrooted network” is used for a fully undirected network. An unrooted network in our sense retains directions on hybrid edges, and thus encodes some information about possible root locations on $N^+$. Figure 1 depicts a topological binary rooted phylogenetic network on the left and its induced topological unrooted network on the right.

For simplicity, when we refer to an *unrooted network* $N^-$ in this paper, either metric or topological, we mean a semidirected network induced from a rooted binary phylogenetic network $N^+$ as in Definition 4. That is, we implicitly assume the existence of $N^+$. This is an important convention to keep in mind, since under the standard graph theoretical definition there are unrooted networks which are not so induced.

Since an unrooted network retains some directed edges, a useful definition of an induced quartet network is more elaborate than the analog for a tree. Recall that a *trek* between vertices *x*, *y* on a network is the union of a semidirected path from some vertex *v* to *x* and a semidirected path from *v* to *y*. A trek is *simple* if the two paths intersect only at *v*.

#### **Definition 5**

Let $N^-$ be a unrooted network on *X*, and let $a,b,c,d\in X$. The *induced quartet network* $Q_{abcd}$ is the unrooted network obtained by

1. Keeping only the edges in simple treks between pairs of elements of $\{a,b,c,d\}$, and then
2. Suppressing all degree two nodes.

In the case that $N^-$ is a metric network, the quartet network $Q_{abcd}$ inherits a metric structure in a natural way: noting that any hybrid edge *e* in $Q_{abcd}$ arises from a single hybrid edge ${\tilde{e}}$ of $N^-$ possibly conjoined with several tree edges, we set the hybridization parameter for *e* equal to that for ${\tilde{e}}$. Edge lengths in $Q_{abcd}$ are simply sums of lengths of conjoined edges from $N^-$.

**Fig. 2:**
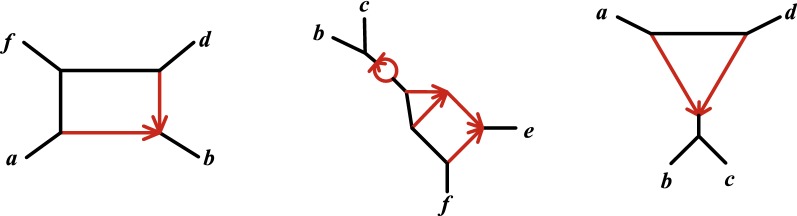
Three quartet networks, $Q_{abdf}$, $Q_{bcef}$, and $Q_{abcd}$, induced from the unrooted network $N^-$ of Fig. 1(R)

Figure 2 shows several quartet networks induced from the unrooted network in Fig. 1.

Finally, most of our results are established only for a subclass of phylogenetic networks exhibiting a *level-1* structure. The definition we give is not the standard one for level-1 (e.g., [14]), but it is equivalent for binary directed networks [15]. We also use our notion of level-1 for the unrooted networks in this paper, where the directions of hybrid edges are preserved.

#### **Definition 6**

Let *N* be a (rooted or unrooted) binary topological network. If no two cycles in the undirected graph of *N* share a vertex, then *N* is *level-1*.

## The network multispecies coalescent model and quartet concordance factors

The *multispecies coalescent model* (MSC) [16, 17] is the standard probabilistic model of incomplete lineage sorting, by which gene trees, showing direct ancestral relationships, form within species trees composed of multi-individual populations. It traces, backwards in time, the lineages of a finite set of individual copies of a gene, sampled from different extant species, as they *coalesce* at common ancestral individuals.

The *network multispecies coalescent model* (NMSC) [18–20] is a generalization of the multispecies coalescent model, which allows a finite number of hybridization events, or other discrete horizontal gene transfer events, between populations. Its parameters are captured by a metric, rooted phylogenetic network, assumed to be binary, as defined above. Branch lengths are given in coalescent units, so that the rate of coalescence between two lineages is 1. At a hybrid node in the network, a gene lineage may pass into either of two ancestral populations, with probabilities given by the hybridization parameters $\gamma , 1-\gamma$ for that node. This differs from other generalizations of the MSC, such as those built on a structured coalescent, where genes may switch populations continuously over an interval in time.

### Quartet concordance factors

The NMSC model is often used to obtain the probability (or density) of observing a specific gene tree (metric or topological, rooted or unrooted) in a species network. The NANUQ algorithm focuses on summaries of gene trees; that is, that a species network produces various gene tree quartets (unrooted topological gene trees on 4-taxa) in parameter-dependent frequencies under the NMSC. The study of these probabilities, and their use for network inference, was pioneered in [1], with further work in [3]. A key concept is that of a quartet concordance factor, whose definition we recall.

A binary unrooted topological tree on four taxa *a*, *b*, *c*, *d* is called a *quartet*, denoted as *ab*|*cd* if deletion of its internal edge gives a connected component $\{a, b\}$. When $n \ge 4$, an *n*-taxon tree *displays* a quartet *ab*|*cd* if the induced unrooted tree on the four taxa is *ab*|*cd*.

#### **Definition 7**

Let $N^+$ be a metric rooted network on a taxon set *X*, and *A*, *B*, *C*, *D* lineages for a single gene sampled from individuals in species $a,b,c,d\in X$ respectively. Given a gene quartet *AB*|*CD*, the *concordance factor* $CF_{AB|CD} = CF_{AB|CD} (N^+)$ is the probability under the NMSC on $N^+$ that a gene tree displays the quartet *AB*|*CD*. The *concordance factor* $CF_{abcd} = CF_{abcd}(N^+)$ is the ordered triple

$$\begin{aligned} CF_{abcd}=(CF_{AB|CD},CF_{AC|BD},CF_{AD|BC}) \end{aligned}$$

of concordance factors of each quartet on the taxa *a*, *b*, *c*, *d*.

When there is no ambiguity, such as when we have a fixed rooted metric network $N^+$ in mind, we denote the concordance factor simply by $CF_{abcd}$. Similarly, when *a*, *b*, *c*, *d* are clear from context (e.g., if $N^+$ has only four taxa), we write *CF* for $CF_{abcd}$. Also, while the language of ‘concordance factor’ is sometimes used for both theoretical values and empirical estimates, in this work we use this term exclusively for the expected values, being careful to refer to ‘estimators of CFs,’ or ‘empirical CFs,’ when these are computed from data.

As established in [1, 3], the concordance factors for a level-1 network $N^+$ depend only on the unrooted metric network $N^-$, and, more precisely, $CF_{abcd}$ depends only on the metric quartet network $Q_{abcd}$ induced from $N^-$. Significantly, these concordance factors carry information about what 4-taxon substructures might be on that network. For instance, if four taxa *a*, *b*, *c*, *d* are related by the tree *ab*|*cd* on $N^-$, then under the NMSC the concordance factors satisfy $CF_{AB|CD} > CF_{AC|BD} = CF_{AD|BC}$. To explain what information $CF_{abcd}$ contains about cycle structure on $N^+$, we quickly review some terminology and results from these works.

**Fig. 3:**
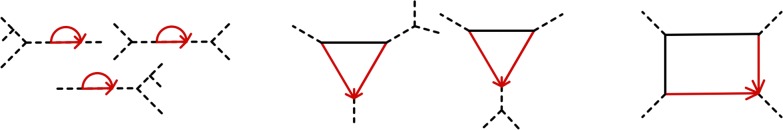
Cycles in a level-1 quartet network are classified as type $m_k$ if they have *m* edges and *k* descendants of the hybrid node. The only cycles possible in a level-1 quartet network are of (L) type $2_1$, $2_2$, and $2_3$; (C) type $3_1$ and $3_2$; and (R) type $4_1$. The dashed lines represent subgraphs that may contain other $m_k$ cycles for $m=2,3$

By an $m_k$-cycle in a level-1 network we mean an *m*-cycle with exactly *k* taxa descended from its unique hybrid node. In a level-1 quartet network, there are exactly 6 types of cycles that may appear: $2_1$-, $2_2$-, $2_3$-, $3_1$-, $3_2$-, and $4_1$-cycles which are depicted in Fig. 3. When considering level-1 quartet networks, there are restrictions on the number and types of cycles that may occur simultaneously. For example, $Q_{abcd}$ might have a $4_1$-cycle or a $3_2$-cycle, but not both.

We next classify concordance factors $CF_{abcd}$ depending on the magnitude of its entries.

#### **Definition 8**

If the two smallest entries of the concordance factor $CF = CF_{abcd}$ are equal, then *CF* is said to be *tree-like*. If a tree-like CF has a unique largest entry, without loss of generality $CF_{AB|CD}$, then *CF* *supports* the quartet *ab*|*cd*. If $CF = (1/3,\, 1/3, \,1/3)$, then it supports all three quartets.

This terminology is motivated by the fact that if a concordance factor CF arises from the NMSC on a species *tree*, then CF is tree-like, and its largest entry indicates the quartet species tree topology [21]. However, as was first shown in [1], certain types of non-tree networks also produce tree-like CFs under the NMSC.

**Fig. 4:**
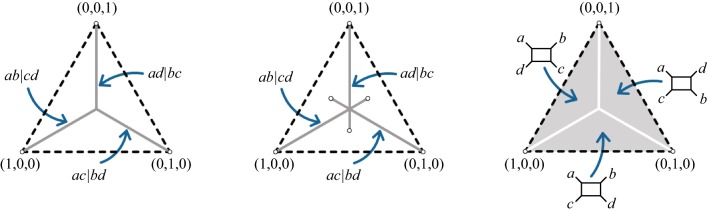
Planar projections of the simplex $\Delta _2$ showing types of concordance factors for networks $N^-_c$ of Proposition 9. (L) Gray line segments represent tree-like CFs that arise from quartet networks with no $3_2$-cycle and with no 4-cycle. (C) Gray line segments represent CFs that arise from quartet networks with a $3_2$-cycle. (R) Gray shaded areas represent CFs that arise from quartet networks containing a 4-cycle. In all three figures, the topology of $N^-_c$ is marked for the appropriate line segments or regions of CFs

Viewing CF as a point in the probability simplex $\Delta _2=\{(x_1,x_2,x_3))\mid x_i\ge 0,\sum x_i=1\}$, as in Fig. 4(L), the tree-like CFs form 3 line segments radiating from the central point (1/3, 1/3, 1/3) to the vertices. With the ordering

$$\begin{aligned} CF_{abcd}=(CF_{AB|CD},CF_{AC|BD},CF_{AD|BC}), \end{aligned}$$

the diagonal segment leading to (1, 0, 0) comprises those CFs supporting *ab*|*cd*, the segment leading to (0, 1, 0) comprises those supporting *ac*|*bd*, and the vertical segment leading to (0, 0, 1) comprises those supporting *ad*|*bc*.

The next proposition summarizes several results from [3]. By *contraction* of a cycle, we mean the removal of its edges followed by the identification of all vertices in it.

#### **Proposition 9**

*Let *$N^+$ *be a level-1 binary quartet network and* $N^-_c$ *the network obtained from* $N^-$ *by contracting all *2*- and 3-cycles and then suppressing degree 2 nodes.*

1. *If *$N^-$* has no cycle of type *$4_1$ *or *$3_2$, *then its concordance factor CF is tree-like, and supports the quartet* $N^-_c$. *That is, if *$N^-_c= ab|cd$, *then*
  $$\begin{aligned} CF_{AB|CD}>CF_{AC|BD}=CF_{AD|BC}. \end{aligned}$$
2. *If *$N^-$ *has a *$3_2$-*cycle, then its concordance factor CF may or may not be tree-like. In particular, CF is on the extended line segment in* $\Delta _2$ *containing the tree-like concordance factors that support the quartet *$N^-_c$. *Specifically, if* $N^-_c= ab|cd$, *then*
  $$\begin{aligned} CF_{AB|CD}\ge 1/6, \text{ and }CF_{AC|BD}=CF_{AD|BC}, \end{aligned}$$
  *and any such tree-like CF supports ab*|*cd*.
3. * If* $N^-$ *has a *$4_1$-*cycle, then its concordance factor CF is not tree-like, and if* $N^-_c$ *displays a 4-cycle joining taxa in circular order a*, *b*, *c*, *d*, *then*
  $$\begin{aligned} CF_{AB|CD}>CF_{AC|BD} \text{ and }CF_{AD|BC}>CF_{AC|BD}.\end{aligned}$$

In Fig. 4, we make concrete the proposition’s results. The CFs for binary quartet networks partition the simplex: $\Delta _2 = \{\text{tree-like CFs}\} \sqcup \{4_1\text{-cycle CFs}\}$, with the collection of CFs for $3_2$-cycles meeting both subsets non-trivially. Notably, if a quartet network $N^-$ has no $3_2$-cycle, then CFs suffice to determine if $N^-_c$ is a tree or a 4-cycle. This idea underlies our algorithm, as well as the network identifiability results from [3].

Indeed, we see from the partition that (in the absence of $3_2$-cycles) the presence of 2-cycles and $3_1$-cycles has no impact on whether a quartet tree or 4-cycle network is supported. This observation leads to the non-identifiability of such cycles on a network by the proof method utilized in [3], and prevents NANUQ from detecting them too. However, since 2- and $3_1$-cycles on a large network model ‘hybridization’ between the most closely related populations (two that split and then rejoin, or hybridization between two populations which have just split from a common one) the inability to infer that such hybridization events occurred by our method may not be too surprising. The SNaQ algorithm [1] is likewise unable to detect these, as it too is based on CFs.

Because concordance factors arising from quartet networks with a $3_2$-cycle (case 2 of Proposition 9) coincide with CFs for particular parameter choices for $4_1$-cycle networks and tree-like networks, such CFs must be handled with delicacy. Clearly, $3_2$-cycles on quartet networks are not identifiable from CFs, and therefore will not be reconstructed by the NANUQ algorithm which focuses only on 4-cycles and tree-like quartet networks. Because such $3_2$-cycles will be disregarded, we investigate them more fully next.

**Fig. 5:**
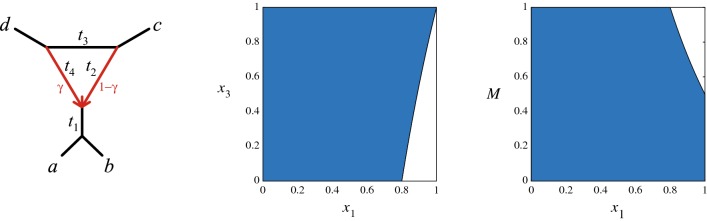
(L) NMSC parameters for an induced unrooted quartet $N^-$ with a $3_2$-cycle. (C) A region of tree-like parameters $(x_1,x_3)$ on $N^-$ for arbitrary $t_2$, $t_4$, $\gamma$. (R) A region of tree-like parameters $(x_1, M)$, where $M = \max \{x_2,x_4\}$ for arbitrary $t_3$, $\gamma$. Transformed parameters are defined by $x_i = e^{-t_i}$

A first observation is that for a tree-like CF arising from a quartet network $N^-$ with a $3_2$-cycle, say with descendants *a*, *b* of the hybrid node as in Fig. 5, then $N^-_c$ has topology *ab*|*cd*. This is exactly the topology supported by the CF, when viewed as arising from a particular parameter choice on the 4-taxon tree *ab*|*cd*. Thus, while determining if the CF arises from a $3_2$-cycle or a tree is not possible, a tree-like CF always correctly supports the topology of $N_c^-$.

This leaves the question of how ‘rare’ are non-tree-like $3_2$-cycle networks, and what metric structure on a $3_2$-cycle network might lead to CFs that coincide with $4_1$-cycle CFs.

### $3_2$-cycles

Let $N^-$ be the unrooted quartet network shown in the left of Fig. 5, with branch length parameters $t_i$ in coalescent units, and hybridization parameter $\gamma$ as shown. With $x_i= e^{-t_i}$ then [1, 3] the quartet concordance factors of $N^-$ are

$$\begin{aligned} CF_{AB|CD}&=\left (1-\gamma \right )^2\left (1-\frac{2}{3}x_1x_2\right )\\& \quad + 2\gamma \left (1-\gamma \right )\left (1-x_1+\frac{1}{3}x_1x_3\right )\\ & \quad + \gamma ^2\left (1-\frac{2}{3}x_1x_4\right ),\\ CF_{AC|BD} & =CF_{AD|BC} =\left (1-\gamma \right )^2\left (\frac{1}{3}x_1x_2\right )\\ & \quad +\gamma \left (1-\gamma \right )x_1\left (1-\frac{1}{3}x_3\right )\\&\quad +\gamma ^2\left (\frac{1}{3}x_1x_4\right ). \end{aligned}$$

We say a choice of parameters $\{t_1,t_2,t_3,t_4,\gamma \}$, or their transformed versions $x_i$, is *tree-like* if the CF for the network is tree-like for those parameters. The set of tree-like parameters for $N^-$ is a region in the 5-dimensional cube, $0\le x_1,x_2,x_3,x_4,\gamma \le 1,$ defined by the polynomial inequality $CF_{AB|CD}\ge CF_{AC|BD}.$

To get a sense of the size of the tree-like region on $N^-$, we sampled uniformly at random $10^{10}$ points in $[0,1]^5$. For untransformed branch length parameters $t_i$, this corresponds to sampling from an exponential distribution with mean 1. We computed that approximately 0.00532 of the resulting CFs were not tree-like. In this sense, non-tree-like CFs from $3_2$-cycles are rare.

For additional insight into tree-like parameters on $N^-$, we investigate CFs as functions of $x_1$ and $x_3$, with $0 < x_2, x_4, \gamma \le 1$, noting that when $x_2$, $x_4$ achieve their maximum value of 1, this corresponds to the network with hybrid branch lengths $t_2 = t_4 = 0$. Concretely, parameters are tree-like if

1 $$\begin{aligned} & CF_{AB|CD} - CF_{AC|BD} \\ & \quad =\,1 - x_1 \, \big ((1-\gamma )^2 x_2+\gamma (1-\gamma ) (3 - x_3) + \gamma ^2 x_4\big ) \\ & \quad \ge \,1 - x_1 \, \big ((1-\gamma )^2 +\gamma (1-\gamma ) (3 - x_3) + \gamma ^2 \big )\nonumber \\ & \quad =1-x_1-\gamma (1-\gamma )x_1(1-x_3) \\ & \quad \ge \,1-x_1-\frac{1}{4} x_1(1-x_3) \, = \, 1 - \frac{1}{4}x_1(5-x_3) \ge 0. \end{aligned}$$

Hence parameters are tree-like for any values of $x_2,x_4,\gamma$ when $x_1 \le 4/(5-x_3)$, a region shown in the center of Fig. 5. This region has area $4 \ln \frac{5}{4} \approx .89$. More crudely, provided $x_1 \le 4/5$ (that is, $t_1 \ge \log (5/4)\approx 0.2231$ coalescent units), then a tree-like CF results regardless of all other parameter values. Thus non-tree-like parameters require that $t_1$ be fairly short, causing substantial incomplete lineage sorting. For comparison, if the internal branch on a rooted 3-taxon species tree has length $t<\log (5/4)$, then fewer than half of the gene trees match the species tree under the MSC.

Although this argument assumed the non-existence of $2_1$-, $2_2$-, and $3_1$-cycles in $N^-$, a general level-1 quartet network with a $3_2$-cycle might have cycles of those types. The result generalizes without difficulty to these more general networks, with $t_1$ the length of the edge descended from the $3_2$-hybrid node. For larger networks, we have the following proposition.

#### **Proposition 10**

*Suppose *$N^+$* is a level-1 network on n taxa and that for each* $m_k$-*cycle with *$m \ge 3$* and *$k \ge 2$* the branch descending from the hybrid node has length *$t \ge \log (5/4)$. * Then under the NMSC model all CFs for induced quartet networks *$\mathcal {Q}$* on *$N^-$* are tree-like, except when *$\mathcal {Q}$* has a *4-*cycle*.

Before proving the proposition, note that an $m_k$-cycle in $N^+$ can induce not only a $4_1$-cycle in an induced quartet network, but also smaller cycles, depending on the particular choice of four taxa. For instance, a 4-cycle in the network of Fig. 1(L) leads to a $3_2$-cycle in the induced quartet network on *a*, *b*, *c*, *d*, as shown in Fig. 2(R).

#### *Proof*

Choose taxa so that the $3_2$-cycle in $N^-_{abcd}$ and its parameters are named as in Fig. 5. Then $t_1 \ge t$ since the edge of length $t_1$ in $N^-_{abcd}$ is made by (possibly) conjoining several edges in $N^+$, including the one of length *t*. The argument following equation (1) now applies. $\square$

The branch length hypotheses in Proposition 10 are sufficient, but not necessary, for tree-like CFs in the presence of $3_2$-cycles. For instance, if a tree edge *e* descendant from a hybrid node in a $3_2$-cycle in $N^+$ is followed by one (or more) 2-cycles, then the length requirement on *e* to produce tree-like CFs might be shortened.

Focusing again on the quartet network of Fig. 5, we now investigate transformed branch length parameters $x_2$, $x_4$ on hybrid edges that lead to tree-like parameter choices. To this end, let $M=\max (x_2,x_4) \le 1$. Then from Eq. (1) for any $x_3$, $\gamma$, we find

$$\begin{aligned} & CF_{AB|CD}- CF_{AC|BD} \\ & \quad \ge 1 - x_1 \, \left ((1-\gamma )^2 M+3 \gamma (1-\gamma ) + \gamma ^2 M\right )\\ & \quad \ge 1-x_1\left( \frac{3+2M}{4}\right) , \end{aligned}$$

and parameters are tree-like if $M\le \min \left( \frac{2}{x_1}-\frac{3}{2}, 1 \right)$, a region shown in blue in Fig. 5(R). Its area is $2 \log (\frac{5}{4} ) + .5 \approx 95\%$ of the $(x_1, M)$-parameter space shown. As a special case, if $M\le \frac{1}{2}$ (equivalently, $\min \{t_2,t_4\}\ge \log (2)\approx 0.693$ coalescent units), parameters are tree-like for all choices of $(x_1, x_3, \gamma )$.

The branch length conditions presented here that rule out non-tree-like $3_2$-cycles come with a caution, since one might prefer to avoid *a priori* modeling assumptions on branch lengths. Nonetheless, our goal has been to suggest that plausible assumptions can rule out non-tree-like CFs arising from $3_2$-cycles in quartet networks. Inspection of empirical CFs from a data set may provide further evidence that no such CFs are involved in a data analysis

## Network split systems and distances

The ability to use quartet CFs to determine whether a quartet network displays a 4-cycle can be combined with ideas from [5] to compute a pairwise distance between taxa on a large *n*-taxon network. Indeed, the intertwining of these ideas with that of a weighted circular split system is the foundation of the NANUQ algorithm. In this section we review the concepts of weighted circular split systems and associated distances, as needed for our inference method.

### Split systems

We adopt standard terminology concerning splits [22]. A *split* $A|B=B|A$ of taxa *X* is a bipartition $X=A\sqcup B$ with *A*, *B* non-empty. The subsets *A*, *B* are called split sets. The set of all splits of *X* is denoted by *Split*(*X*), and $\mathcal {S}\subseteq Split(X)$ is called a *split system* on *X*.

#### **Definition 11**

A split system $\mathcal {S}\subseteq Split(X)$ is *circular* if there exists a linear ordering $x_1< \cdots <x_n$ of the elements of *X* such that each split in *S* has the form *A*|*B* with

$$\begin{aligned} A=\{x_p,x_{p+1},....,x_{q-1},x_q\} \end{aligned}$$

for appropriately chosen $1\le p< q< n$. The ordering of the $x_i$ is a *circular ordering* for $\mathcal {S}$.

A circular ordering for $\mathcal {S}$ is not unique, since it can be modified by cyclically permuting the $x_i$ (e.g., replaced with $x_2<x_3<\dots<x_n<x_1$) or by inversion (replaced with $x_n<x_{n-1}<\dots<x_1$), while remaining a circular ordering for $\mathcal {S}$. We treat such variants as the same, without further comment.

Given a tree *T* on *X*, deleting an edge defines a split according to the connected components of the resulting graph. The set of all such displayed splits is denoted $\mathcal {S}(T)$, and it is clear from a planar depiction of a tree that $\mathcal {S}(T)$ is circular.

For a tree, the correspondence between edges and displayed splits allows edge weights to be viewed as split weights, by setting weights of non-displayed splits to 0. This is a special case of a *weighted split system on X*, a map

$$\begin{aligned} \omega : Split(X)\rightarrow \mathbb {R}^{\ge 0}. \end{aligned}$$

A weighted split system $\omega$ on *X* induces a distance function $d_\omega$ on *X* by

$$\begin{aligned} d_\omega (x,y)=\sum _{s\in S_{xy}} \omega (s), \end{aligned}$$

where $S_{xy}\subseteq Split(X)$ is the set of splits separating *x* and *y*, i.e., splits *A*|*B*, with $x\in A$ and $y\in B$. Clearly $d_\omega$ is non-negative valued, with $d_\omega (x,x)=0$, $d_\omega (x,y)=d_\omega (y,x)$.

Recall that the support of a weighted split system, denoted ${\text{supp}}(\omega )$, is the set of splits on which $\omega$ is non-zero.

#### **Definition 12**

A weighted split system $\omega$ on *X* is said to be *circular* if ${\text{supp}}(\omega )$ is circular. A distance function *d* on *X* is said to be *circular* if $d=d_\omega$ for some circular weighted split system $\omega$.

As pointed out in [22], it follows from [23] that a circular distance function *d* uniquely determines the weighted split system $\omega$ such that $d= d_\omega$.

### Splits from unrooted networks

Our notion of splits associated to a network, and some related terminology, is not standard, but is essential to this work. In particular, we focus only on phylogenetic unrooted networks $N^-$ as in Definition 4, where $N^-$ is induced from a rooted phylogenetic network and the direction of hybrid edges are retained in $N^-$.

#### **Definition 13**

Let $N^-$ be a unrooted network on *X*. An unrooted tree *T* on *X* is *displayed* on $N^-$ if it can be obtained from $N^-$ by deleting some edges, including at least one hybrid edge from each pair, undirecting remaining hybrid edges, and suppressing degree 2 nodes. The set of all unrooted topological trees on *X* displayed on $N^-$ is called the *grove* of $N^-$, denoted $\mathcal {G}(N^-)$.

If $N^-$ has an *m*-cycle with $m\ge 4$, then the grove $\mathcal {G}(N^-)$ is a proper subset of the displayed trees on the undirected network $N^u$ underlying $N^-$ as defined in [13]. This is because $N^u$ is obtained by undirecting the hybrid edges in $N^-$, and there is additional freedom in the choice of edges to delete in $N^u$ to obtain its displayed trees: It is not necessary to delete at least one of the edges from $N^u$ that arose from each pair of hybrid edges in $N^-$.

If $N^-$ has 2- or 3-cycles, then deleting either hybrid edge in those cycles yields trees with the same topology, and hence gives the same elements of $\mathcal {G}(N^-)$. In contrast, for cycles of size 4 or larger, the trees in $\mathcal {G}(N^-)$ vary with the choice of hybrid edge deleted. Since we assume that $N^-$ is level-1 with *k* cycles of size $\ge 4$, then $|\mathcal {G}(N^-)|=2^k$.

#### **Definition 14**

For an unrooted network $N^-$, the set of splits

$$\begin{aligned}\mathcal {S}(N^-)=\bigcup _{T\in \mathcal {G}(N^-)} \mathcal {S}(T)\end{aligned}$$

is called the *(unweighted) split system for* $N^-$. A *weighted split system for* $N^-$ is any weighted split system with support $\mathcal {S}(N^-)$.

The study of undirected networks in [13] provides the following theorem, establishing a connection between circular split systems and undirected level-1 networks.

#### **Theorem 15**

([13]) *Let S be a split system on a set X*.* Then S is circular if, and only if, there exists an undirected level-1 network N such that *$S \subseteq \mathcal {S}(N)$,* the set of all splits of all trees on X* *displayed on N*.

Note that if $N^u$ is the undirected network underlying the unrooted network $N^-$, then $\mathcal {S}(N^-) \subseteq \mathcal {S}(N^u)$. As a consequence, we obtain the following.

#### **Corollary 16**

*If* $N^-$* is a level-1 unrooted network, then* $\mathcal {S}(N^-)$* is circular.*

## Quartet distance for level-1 networks

As shown in [5], a topological tree has a natural metrization tied to the quartets displayed on the tree. Importantly, intertaxon distances from this metrization can be computed from the collection of displayed quartets, without having knowledge of the full tree, giving a means for consistently inferring the tree topology. After briefly reviewing these results in the tree setting, we generalize them to the setting of level-1 networks.

### Quartet distance on a tree

For an unrooted binary topological phylogenetic tree *T* on *X*, any internal edge *e* induces a partition of *X* into 4 non-empty blocks, $X_1$, $X_2$, $X_3$ and $X_4$, where the split associated to *e* is $s_e=X_1\cup X_2|X_3\cup X_4$, and the splits associated to the 4 adjacent edges have an $X_i$ as one split set. Similarly, a pendant edge *e* to taxon *a* induces a partition into 3 blocks $X_1$, $X_2$ and $\{a\}$, where $s_e=\{a\}|X_1\cup X_2$, and the splits associated to the 2 edges adjacent to *e* have an $X_i$ as one split set. The quartet weight function $w_T: Split(X) \rightarrow {\mathbb {R}}$ is defined as

$$\begin{aligned} w_T(s)={\left\{ \begin{array}{ll} |X_1||X_2|+|X_3||X_4| &{} \text{ if }s=s_e,\, e \text{ internal}, \\ |X_1||X_2| &{} \text{ if }s=s_e,\,e\text{ pendant}, \\ 0 &{} \text{ if }s\text{ is not on }T. \end{array}\right. } \end{aligned}$$

This split weight function then induces $d_{w_T}$, the quartet distance function on *X*. This distance is a tree metric, and therefore can be used to reconstruct the topological binary *n*-taxon tree *T* by several algorithms. Significantly, the distance function $d_{w_T}$ can computed another way, from the set of quartets displayed on *T*, without prior knowledge of the full tree topology.

#### Theorem 17

[5] * For any quartet q on taxa in X with *$|X|=n$, * let *$\rho _{xy}(q)=1$* if *$q=xz|yw$,* and 0 otherwise. That is,* $\rho _{xy}$* is the indicator function for separation of x and y* *on a quartet. Then for an unrooted binary tree T on X*,* and any* $x,y\in X$,

2 $$\begin{aligned} d_{w_T}(x,y)=2\sum _{{q\text{o}n }T}\rho _{xy}(q)+2n-4. \end{aligned}$$

### Quartet distance on a network

To generalize Theorem 17 to a network, we begin with a definition.

#### Definition 18

Let $N^-$ be an unrooted network on *X*. Then the *quartet weight function* $\omega _{N^-}$ is defined by

$$\begin{aligned} \omega _{N^-}(s) = \sum _{T\in \mathcal {G}(N^-)} w_T(s), \end{aligned}$$

where $s \in Split(X)$ and $w_T(s)$ is the quartet weight function on *T*.

Note that since ${\text{supp}}(w_T)=\mathcal {S}(T)$ for each *T*, ${\text{supp}}(\omega _{N^-})=S(N^-)$. Thus, by Corollary 16, the quartet weight function $\omega _{N^-}$ is a weighted circular split system for $N^-$. Moreover, the induced distance function is easily related to those for the trees in the grove $\mathcal {G}(N^-)$.

#### **Lemma 19**

*Let *$N^-$* be a level-1 unrooted network on X*.* Then*

$$\begin{aligned} d_{\omega _{N^-}}=\sum _{T\in \mathcal {G}(N^-)}d_{w_T}. \end{aligned}$$

#### *Proof*

For $x,y\in X$, let $S_{xy}\subset Split(X)$ be the set of splits separating *x* and *y*. Then

$$\begin{aligned}d_{\omega _{N^-}}(x,y) & =\sum _{s\in S_{xy}}\omega _{N^-}(s) =\sum _{s\in S_{xy}} \sum _{T\in \mathcal {G}(N^-)} w_T(s)\\ & =\sum _{T\in \mathcal {G}(N^-)} \sum _{s\in S_{xy}} w_T(s) =\sum _{T\in \mathcal {G}(N^-)}d_{w_T}(x,y). \end{aligned}$$

$$\square$$

To state a network analog of Theorem 17, we must extend the indicator function $\rho _{xy}$ to quartet networks.

#### **Definition 20**

Let $Q_{xyzw}$ be an unrooted level-1 4-taxon network on 4 distinct taxa $x,y,z,w\in X$. After contracting all 2- and 3-cycles, and suppressing degree 2 nodes, we obtain a network ${\widetilde{Q}}_{xyzw}$ that is either a tree or has a single 4-cycle. Let

$$\begin{aligned} \rho _{xy}( Q_{xyzw})={\left\{ \begin{array}{ll} 0 & \text{ if }{\widetilde{Q}}_{xyzw}\text{ has form }xy|zw,\\ 1/2 & \begin{aligned} & \text{if }{\widetilde{Q}}_{xyzw}\text{ has a 4-cycle}\\ & \text{ with }x,y\text{ adjacent, }\end{aligned}\\ 1 & \text{ otherwise}. \end{array}\right. } \end{aligned}$$

**Fig. 6:**
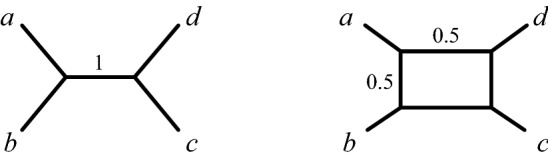
For the tree $Q_{abcd}$ on the left, $\rho _{ab}( Q_{abcd})=0$ and $\rho _{ac} ( Q_{abcd} ) = 1$, since *a* and *c* are separated by *ab*|*cd*, but *a* and *b* are not. For the quartet network $Q_{abcd}$ on the right, $\rho _{ab}( Q_{abcd})=1/2$ and $\rho _{ac}( Q_{abcd})=1$, since the trees displayed by $Q_{abcd}$ are *ab*|*cd* and *ad*|*bc*

In the case $Q_{xyzw}$ is a tree, this definition agrees with that in Theorem 17. An intuitive way of viewing this extension to networks is to observe that when ${\widetilde{Q}}_{xyzw}$ is a 4-cycle, $\rho _{xy} (Q_{xyzw})$ is the average of the values of $\rho _{xy} (T)$ for $T \in \mathcal {G} ({\widetilde{Q}}_{xyzw})$, so $\rho _{xy}$ measures how separated *x* and *y* are on $Q_{xyzw}$. See Fig. 6.

#### **Lemma 21**

*For an unrooted level-1 network *$N^-$,* with k cycles of size *$\ge 4$,* and distinct *$x,y,z,w\in X$,* let *$Q_{xyzw}$* be the induced unrooted 4-taxon network on x*, *y*, *z*, *w*.* Then*

$$\begin{aligned} \rho _{xy}( Q_{xyzw})=\frac{1}{2^k}\sum _{T\in \mathcal {G}(N^-)}\rho _{xy}(T_{xyzw}). \end{aligned}$$

#### *Proof*

If $\rho _{xy}( Q_{xyzw})=0$, then there is no $T\in \mathcal {G}(N^-)$ with $T_{xyzw}$ separating *x*, *y*, so the equation holds. If $\rho _{xy}( Q_{xyzw})=1/2$, then ${\widetilde{Q}}_{xyzw}$ has two hybrid edges, which are induced from hybrid edges of $N^-$. Each of these is deleted in exactly half of the $2^k$ trees in $\mathcal {G}(N^-)$, so $2^{k-1}$ of the $T\in \mathcal {G}(N^-)$ have $T_{xyzw}$ displaying a quartet separating *x*, *y*, and the equation holds. Finally, if $\rho _{xy}( Q_{xyzw})=1$, so ${\widetilde{Q}}_{xyzw}$ is either a quartet tree separating *x*, *y*, or has a 4-cycle with *x*, *y* opposite in its circular ordering, then for all $T\in \mathcal {G}(N^-)$, $T_{xyzw}$ will display a quartet separating *x*, *y*, so the equation holds. $\square$

We now define a distance function in terms of quartet networks displayed on the network.

#### **Definition 22**

Let $N^-$ be an unrooted level-1 network on *X*. Then the *quartet distance* $d_{Q,N^-}$ is

$$\begin{aligned} d_{Q,N^-}(x,y)=2\sum _{z,w\ne x,y}\rho _{xy}(Q_{xyzw})+2n-4, \end{aligned}$$

with $x,y \in X$, distinct from $w,z \in X$.

Note that if $N^-=T$ is a tree, the definition of $d_{Q,N^-}(x,y)$ agrees with Eq. (2). We now prove the network analog of Theorem 17, showing that the network distance $d_{\omega _{N^-}}$ can be computed from induced quartet networks.

#### **Theorem 23**

*Let *$N^-$* be an unrooted level-1 network on X*,* with k cycles of size *$\ge 4$.* Then*

$$\begin{aligned} d_{\omega _{N^-}}={2^k}d_{Q,N^-}. \end{aligned}$$

#### *Proof*

Using Lemma 19, Theorem 17, and Lemma 21, for $x\ne y\in X$,

$$\begin{aligned} d_{\omega _{N^-}}(x,y) & =\sum _{T\in \mathcal {G}(N^-)} d_{w_T}(x,y)\\&=\sum _{T\in \mathcal {G}(N^-)} \left( 2 \sum _{q\text{ on }T}\rho _{xy}(q) +2n-4\right) \\&=2\sum _{T\in \mathcal {G}(N^-)} \sum _{q\text{ on }T} \rho _{xy}(q) \ +2^k(2n-4) \\& =2\sum _{T\in \mathcal {G}(N^-)} \sum _{z,w\ne x,y} \rho _{xy}(T_{xyzw}) \ +2^k(2n-4) \\& =2 \sum _{z,w\ne x,y} \sum _{T\in \mathcal {G}(N^-)} \rho _{xy}(T_{xyzw}) \ +2^k(2n-4) \\& =2 \sum _{z,w\ne x,y} 2^k \rho _{xy}(Q_{xyzw}) \ +2^k(2n-4) \\& ={2^k} \, d_{Q,N^-}(x,y). \end{aligned}$$

$$\square$$

The import of this theorem is that from the induced quartet networks on $N^-$ we can compute the distance $d_{Q,N^-}$, which is, up to scaling, $d_{\omega _{N^-}}$, the distance from a weighted split system. In contrast, computing $d_{\omega _{N^-}}$ directly from definition requires knowing $\mathcal {G}(N^-)$, the collection of trees on *X* displayed on $N^-$. This lies at the heart of our algorithm for network inference under the NMSC, as we can obtain information about induced quartet networks from biological data relatively easily, using empirical concordance factors, while information about the trees displayed on the species network does not seem to be directly obtainable.

Furthermore, since by Corollary 16 the underlying quartet weighted split system is circular, we have the following.

#### **Corollary 24**

*Let *$N^-$* be an unrooted level-1 network. Then the distance* $d_{Q,N^-}$* arises from a weighted circular split system, with support *$\mathcal {S}(N^-)$.

Thus given sufficient information on induced quartet networks to compute $d_{Q,N^-}$, even approximately as in the presence of error, methods for analyzing distances from weighted circular split systems, such as the NeighborNet algorithm, can be productively applied, as we show in the next section.

## Splits graphs from the network quartet distance

The last sections have shown a path toward obtaining, under the NMSC model, the distance associated to the weighted circular split system $\omega _{N^-}$. But for this to have value, we need to be able to extract from this distance information about features of $N^-$. While there is a well developed theory of splits graphs [7, 11, 23, 24], associated to distances from such split systems, and splits graphs are networks, one can not hope that such splits graphs give $N^-$ directly. In particular splits graphs have no directed edges, and are generally not level-1.

Our goal in this section is thus to investigate the relationship between a level-1 network and the splits graphs obtainable from the quartet distance for that network. We develop precise rules by which one can interpret features in a splits graph for $\omega _{N^-}$ to obtain much information on the topological features of $N ^-$. While there is some overlap between the results in this section and those of [12], we give a complete presentation as is necessary for our more detailed results.

The tree edges (i.e., the undirected edges) in a level-1 unrooted network $N^-$ can be classified into two types, extending Definition 1 in this setting. Specifically, a *cycle edge* in $N^-$ is an undirected edge in a cycle, and a *cut edge* is an undirected edge that is not a cycle edge. Any *k*-cycle in $N^-$ is then composed of $k-2$ cycle edges and 2 hybrid edges.

These notions extend to trees displayed on networks. For any $T\in \mathcal {G}(N^-)$, the edges of *T* arise from those of $N^-$ in one of the following ways:

1. An edge ${\bar{e}}$ of *T* is obtained directly from an edge of $N^-$. Then ${\bar{e}}$ is called a cycle or cut edge of *T* according to its classification in $N^-$.
2. An edge ${\bar{e}}$ of *T* is obtained from several edges of $N^-$ by suppressing internal nodes of degree 2. Since $N^-$ is level-1, at least one of these conjoined edges of $N^-$ is a cut edge, so we refer to ${\bar{e}}$ as a cut edge of *T*.

As we show below, cut edges in $N^-$ correspond to splits $s \in \mathcal {S}(N^-)$ that occur on every $T \in \mathcal {G}(N^-)$, while a split ${\bar{s}}$ derived from a cycle edge on *T* does not occur on every $T^\prime \in \mathcal {G}(N^-)$. Moreover, we see that edges in 2-cycles and 3-cycles on $N^-$ induce *only* cut edges on any $T\in \mathcal {G}(N^-)$. For $k\ge 4$, a *k*-cycle on $N^-$ will induce $k-3$ cycle edges on any $T\in \mathcal {G}(N^-)$, since one hybrid edge is deleted, one hybrid edge is conjoined with its descendent cut edge, and one cycle edge is conjoined with a cut edge.

A split $s\in \mathcal {S}(N^-)$ is called a *cycle split* (respectively, a *cut split*) if $s=s_{{\bar{e}}}$ for a cycle edge (respectively, a cut edge) ${\bar{e}}$ on some $T\in \mathcal {G}(N^-)$. Note that the cut splits are precisely those splits obtained from $N^-$ by deletion of a cut edge, and that these two classes of splits form a partition of $\mathcal {S}(N^-)$.

In the next lemma, we prove that the quartet weight function $\omega _{N^-}$ on an unrooted network $N^-$ carries no information about 2- or 3-cycles.

### **Lemma 25**

*Let *$N^-_c$* be the graph obtained from a level-1 binary network* $N^-$* by contracting each 2- and 3-cycle to a vertex and then suppressing degree 2 nodes. Then *$\omega _{N^-_c}=\omega _{N^-}$.

### *Proof*

If one or the other hybrid edge in a 2- or 3-cycle on $N^-$ is deleted, the resulting network has the same topology as obtained by contracting the cycle. Thus $N^-$ and $N^-_c$ display the same topological trees. $\square$

In the next lemma, we formalize some observations made above.

### Lemma 26

*Let *$s\in \mathcal {S}(N^-)$* for a level-1 binary network *$N^-$.* Then the following are equivalent:*

1. $$s\in \mathcal {S}(T)$$* for all *$$T\in \mathcal {G}(N^-)$$,
2. *On every *$T\in \mathcal {G}(N^-)$* there is a cut edge *${\bar{e}}$* such that *$s=s_{{\bar{e}}}$,
3. *s is compatible with every *$s^\prime\in \mathcal {S}(N^-)$.

### *Proof*

Clearly (2) implies (1). To see that (1) implies (2), suppose on some tree $T\in \mathcal {G}(N^-)$ there is a cycle edge ${\bar{e}}$ with $s=s_{{\bar{e}}}$. Then ${\bar{e}}$ arises from a cycle edge in $N^-$ and that cycle has hybrid edges $e_1$ and $e_2$, where $e_1$ was deleted to form *T*. Then no tree $T^\prime\in \mathcal {G}(N^-)$ which is formed by deleting $e_2$ will display *s*. This contradicts (1).

That (1) implies (3) is immediate. For the converse, observe that since $N^-$ is binary, each $T\in \mathcal {G}(N^-)$ is binary. But the set of splits on a binary tree is maximal with respect to compatibility, so (3) implies (1). $\square$

The equivalences in Lemma 26 imply that a split from a cycle edge in some $T\in \mathcal {G}(N^-)$ is incompatible with some split from a cycle edge on some other tree in $\mathcal {G}(N^-)$, an observation we further refine in the following lemma.

### Lemma 27

*Let *$s,s^\prime\in \mathcal {S}(N^{-})$* for a level-1 binary network *$N^-$.* Then* $s,s^\prime$* are incompatible if, and only if, there are cycle edges* $e,e^\prime$* (not necessarily distinct) on *$N^{-}$* in the same cycle C*, * and *$T,T^\prime \in \mathcal {G}(N^-)$* such that *$e,e^\prime$* induce cycle edges *${\bar{e}}, {\bar{e}}^\prime$* on *$T,T^\prime$* with *$s=s_{{\bar{e}}}, s^\prime=s_{{\bar{e}}^\prime}$* and* $T,T^\prime$ *were obtained by deleting different hybrid edges from C*.

### Proof

Consider incompatible $s,s^\prime\in \mathcal {S}(N^{-})$. Then by Lemma 26, there exist $T,T^\prime\in \mathcal {G}(N^-)$ with cycle edges ${\bar{e}}, {\bar{e}}^\prime$ where $s=s_{{\bar{e}}}, s^\prime=s_{{\bar{e}}^\prime}$. The edges ${\bar{e}}, {\bar{e}}^\prime$ are induced from cycle edges $e, e^\prime$ in $N^-$.

Suppose $e,e^\prime$ are in cycles $C\ne C^\prime$. Now *T* determines a hybrid edge of *C* whose removal from $N^-$, along with the removal of *e*, determines the split *s*, and $T^\prime$ similarly determines a hybrid edge of $C^\prime$. Removing these two hybrid edges, together with one hybrid edge from every other cycle on $N^-$ determines a tree $T^{\prime\prime}\in \mathcal {G}(N^-)$. But $T^{\prime\prime}$ has both $s,s^\prime$ as displayed splits, which implies they are compatible. Thus $e,e^\prime$ must be in the same cycle on $N^-$.

Moreover, $T,T^\prime$ must be obtained by deleting different hybrid edges in the cycle containing $e,e^\prime$, since if the same hybrid edge were deleted, the splits $s,s^\prime$ would again be displayed on a common tree, and hence be compatible.

For the converse, suppose $e,e^\prime$ are cycle edges in cycle *C* of $N^-$, which induce cycle edges in trees $T,T^\prime\in \mathcal {G}(N^-)$, where $T,T^\prime$ are obtained by deleting different hybrid edges in *C*. Let $X=X_0 \sqcup X_1\sqcup X_2 \sqcup \dots \sqcup X_{m-1}$ be the partition of *X* obtained from the connected components of the graph resulting from removing all edges of *C* from $N^-$. Suppose further that the ordering of these sets reflects the ordering around the cycle, so that $X_0$ is descendants of the hybrid node, and $X_1,X_{m-1}$ are its neighbors, etc. Then, without loss of generality, we may assume that split $s_e$ displayed on *T* is $X_0\cup \dots \cup X_k| X_{k+1}\cup \dots \cup X_{m-1}$ with $1\le k\le m-3$, while the split $s_{e^\prime}$ displayed on $T^\prime$ is $X_0\cup X_{m-1}\cup \dots \cup X_{\ell +1}| X_{\ell }\cup \dots \cup X_1$ with $2\le \ell \le m-2$. These splits are incompatible as claimed. $\square$

Split networks [11], also known as splits graphs, provide a valuable visual tool for interpreting split systems. In what follows, we use the terminology ‘splits graph’ exclusively to avoid confusion with the species networks $N^+$ and $N^-$ associated with the NMSC.

In a splits graph, each edge is colored by exactly one of the splits, with each split possibly coloring multiple edges. Deleting all edges with a common color leaves two connected components, with taxon labels on the components giving the split sets. Unfortunately splits graphs are generally not uniquely determined by split systems. However, since the split systems of interest here arise from level-1 networks $N^-$, and thus are circular by Corollary 16, we can impose an additional requirement, that of ‘frontier-minimality’ developed below, to determine most features of $N^-$ from interpretation of a frontier-minimal splits graph. The Circular Network Algorithm of [7] is the key to both showing split graphs with this additional property exist in this case, and producing them in specific instances.

Recall that the *frontier* of a planar graph is the subset of edges adjacent to the unbounded component of its complement in the plane (more informally, the “outside” edges of the graph). A graph is *outer-labelled* if the labelled vertices are in the frontier. Also, a *blob* on a network is a maximal set of edges in undirected edge-intersecting cycles. On an unrooted level-1 network such as $N^-$, a blob is simply an undirected version of a cycle.

### **Lemma 28**

*Let *$S=S_c \sqcup S_i$* be a circular split system, with *$S_c$* the subset of splits compatible with all others in S*,* and *$S_i$* those incompatible with at least one other. Then the Circular Network Algorithm*   *of*  [7]  *produces an outer-labelled planar splits graph*  $N_S$* such that*

1. *If *$s\in S_c$,* then s colors exactly one edge in the frontier of *$N_S$,* and this edge is not in any blob.*
2. *If *$s\in S_i$,* then s colors precisely 2 edges in the frontier (and possibly additional edges not in the frontier) which lie in the same blob.*
3. *If *$s,s^\prime \in S_i$* are incompatible, then they color frontier edges in the same blob.*

### *Proof*

The Circular Network Algorithm works iteratively, by adding new vertices and edges as each split is considered in some order, to produce an outer-labelled splits graph [7].

We may assume the trivial splits are in the system. The algorithm begins with these splits represented by a star tree, and the stated properties hold. Each time an additional split *s* is considered, the algorithm first determines if this split is incompatible with the current graph $G_i$. If it is, the algorithm ‘duplicates’ parts of the frontier, composed of some edges labelled by splits incompatible with *s*, joining the duplicated section to the old part by ‘ladder’ edges colored by the new split *s* to form $G_{i+1}$. This makes the frontier grow by 2 edges colored by *s*, and ensures that any splits incompatible with *s* previously coloring only one frontier edge in $G_i$, now color two frontier edges in $G_{i+1}$. Then any two edges colored by the same split lie in the same blob, as do frontier edges coloring incompatible splits.

If the new split $s \in S_c$, then, reminiscent of the tree-popping algorithm, a single new edge in $G_i$ is introduced to form $G_{i+1}$ and is colored by *s*. This new edge is not in a blob. $\square$

This coloring of edges in the frontier of the splits graph produced by the Circular Network Algorithm can be characterized in an alternative, less algorithmic, way.

### **Definition 29**

If *S* is a circular split system on *X*, then an outer-labelled planar splits graph $N_S$ on *S* is *frontier-minimal*, if $N_S$ contains the minimal number of frontier edges among all outer-labelled planar splits graphs on *S*.

### **Proposition 30**

*Any frontier-minimal splits graph *$N_S$* for a circular split system* *S* *has properties (1), (2), and (3) of Lemma*  28. * Moreover, the Circular Network Algorithm*   *produces a frontier-minimal splits graph.*

### *Proof*

First, observe that each split in *S* must label at least one frontier edge, else deletion of edges labelled by that split would not disconnect $N_S$.

Next, recall that the operation of contraction of a split *s* in a splits graph for $\mathcal {S}$, which identifies the two endpoints of each edge labelled by *s* and deletes the edge, yields a splits graph for $\mathcal {S}\smallsetminus \{s\}$ (Lemma 5.10.1 of [11]). Moreover, frontier edges resulting from contraction must arise from frontier edges in the original splits graph. If $s, s^\prime\in S_i$ are incompatible splits in a splits graph for $\mathcal {S}$, then by contracting all other splits we obtain a split network depicting only these two. Now if it were the case that only one frontier edge in this splits graph were labelled by *s*, deletion of that edge must separate the graph. But then, since $s^\prime$ is incompatible with *s*, $s^\prime$ must label edges whose deletion disconnects each of the components obtained by deleting the *s* edge. But this implies that deleting only the $s^\prime$ edges in $N_S$ separates the graph into at least 3 components, which contradicts that it is a splits graph. Thus *s* labels at least 2 frontier edges.

It follows that any splits graph has at least $|S_c| +2|S_i|$ frontier edges, and since this minimal count is achieved by the splits graph output from the Circular Network Algorithm, a frontier-minimal splits graph has $|S_c| +2|S_i|$ frontier edges.

Furthermore, in any splits graph for *S* each element of $S_i$ colors at least two frontier edges and each element of $S_c$ at least one. It then follows from the count of frontier edges in a frontier-minimal splits graph that the elements of $S_i$ color precisely two frontier edges, and elements of $S_c$ precisely one. The single frontier edge labelled by an element of $S_c$ cannot lie in a blob, since otherwise deleting it would not disconnect the graph. This establishes properties (1) and (2) of Lemma 28.

Finally, if $s\in S_i$, then for any $s^\prime\in S_i$ incompatible with *s*, contracting all splits but $s,s^\prime$ in a frontier-minimal splits graph must give a splits graph with four frontier edges. By considering all possible such graphs, these edges must form a 4-cycle with edges labelled in order $s,s^\prime,s,s\prime$. Since these four edges are in the same blob on this graph, they must be in the same blob in the original graph. $\square$

In [7] it is shown that the Circular Network Algorithm produces a splits graph minimal in a different sense: It has the smallest number of edges among all splits graphs whose bounded faces are parallelograms (i.e., quadrilaterals with opposite sides sharing colors). This addresses internal structure of the blobs, which our notion of frontier-minimal ignores. We have not investigated whether the two notions of minimality are equivalent, nor to what extent a frontier-minimal splits graph for a circular split system is unique.

The *tree of blobs* of a graph is the graph obtained by contracting edges and vertices in each blob to a single vertex.

### **Corollary 31**

*The tree of blobs of a level-1 network *$N^-$* is isomorphic to the tree of blobs of a frontier-minimal splits graph for *$\mathcal {S}(N^-)$.

### *Proof*

The tree of blobs of $N^-$ displays precisely those splits associated to cut edges of $N^-$. By Lemma 26, these are precisely the splits compatible with all others in $\mathcal {S}(N^-)$, and by Proposition 30, the tree of blobs of a frontier-minimal splits graph displays the same set. $\square$

**Fig. 7:**
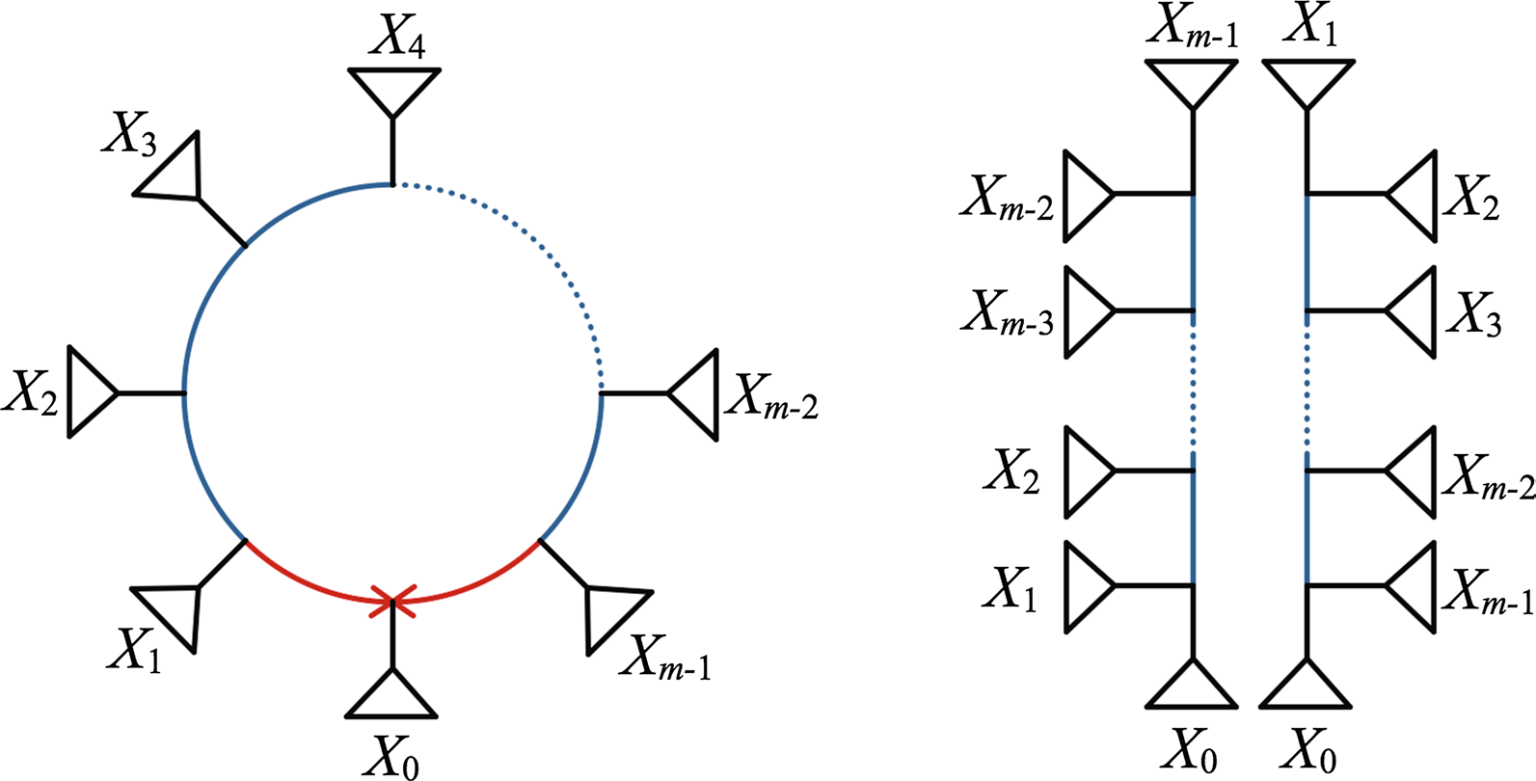
(L) A cycle in a level-1 network $N^-$, and (R) the two simpler networks produced from it by deleting one hybrid edge. The cycle edges in these networks that arise from the original cycle are shown in blue. If $N^-$ has a single cycle, then the networks on the right are the two trees in $\mathcal {G}(N^-)$

To go further, we investigate how the structure of a blob (a cycle) in $N^-$ corresponds to a related structure of a blob (*not* generally a cycle) in a frontier-minimal splits graph for $\mathcal {S}(N^-)$. The following, which characterizes splits associated to a cycle in $N^-$, follows straightforwardly from definitions, so a formal proof is omitted. The argument is readily supplied by considering Fig. 7, which depicts a single cycle in $N^-$, and the two networks obtained from it by deleting one or the other hybrid edge.

### **Lemma 32**

*Suppose a level-1 unrooted network *$N^-$* has k cycles of size *$\ge 4$. * Let C be an m*-*cycle on *$N^-$, $m\ge 4$, *and *$X=X_0 \sqcup X_1\sqcup X_2 \sqcup \dots \sqcup X_{m-1}$* the partition of X obtained from the connected components of the graph resulting from removing all edges of C from *$N^-$. * Suppose further that the ordering of these sets reflects the ordering around the cycle, so that *$X_0$* is the descendants of the the hybrid node, and* $X_1,X_{m-1}$ *are its neighbors, etc. (see Fig.* 7*). Then the cycle splits in *$\mathcal {S}(N^-)$* arising from edges in* *C are*

3 $$\begin{aligned}&X_0\cup X_1\cup {\cdots} \cup X_i| X_{i+1}\cup {\cdots} \cup X_{m-1},\nonumber \\&\quad \qquad 1\le i\le m-3, \end{aligned}$$

4 $$\begin{aligned}&X_0\cup X_{m-1} \cup {\cdots} \cup X_{j+1}| X_{j}\cup {\cdots} \cup X_1,\nonumber \\&\quad \qquad 2\le j\le m-2, \end{aligned}$$

*all with *$\omega _{N^-}(s)= 2^{k-1}$.* Those splits of the form *  (3)* (respectively *(4)*) are compatible with all others of that form. Splits of the form *(3)* are incompatible with those of the form *(4).* Splits of the form *(3)* or *(4)* are compatible with all other elements of *$\mathcal {S}(N^-)$.

*Moreover,* $(X_0,X_1,X_2,\dots , X_{m-1})$* is the only circular ordering of the* $X_i$* consistent with these splits, and with* $X_m=X_0$* the number of cycle splits arising from C that separate* $X_i$*  from* $X_{i+1}$* is*

$$\begin{aligned}{\left\{ \begin{array}{ll} m-3 & \text{ if }i=0,m-1,\\ 1& \text{ if }i=1,m-2,\\ 2& \text{ otherwise.} \end{array}\right. } \end{aligned}$$

The next lemma describes the part of the frontier in a frontier-minimal splits graph arising from splits associated to a single *m*-cycle, a description which will be used later to identify hybrid edges.

### **Lemma 33**

*With notation as in Lemma* 32, *a frontier-minimal splits graph for the cycle splits *$\mathcal {S}(C)$* arising from a single cycle C of size *$m\ge 4$* in *$N^-$* forms a single blob whose frontier is a cycle of size *$4(m-3)$.* Moreover, there are distinct vertices labelled in circular order by *$X_0,X_1,\dots , X_{m-1}$* along the frontier, with the number of edges between labels *$X_i, X_{i+1}$* equal to the number of splits in S*(*C*)* that separate* $X_i,X_{i+1}$.

### *Proof*

Consider two splits associated to the cycle. By Lemma 32, they are either incompatible, or they are both incompatible with a third split from the same cycle. By Lemma 28, they therefore color edges in the same blob, and it follows that there is only one blob in the splits graph. Since by Lemma 32 there are $2(m-3)$ splits associated to the cycle, by Proposition 30 the blob has $|S_c| +2|S_i| = 4(m-3)$ edges in its frontier.

Also by Lemma 32 there exist splits separating any $X_i,X_j$, $i\ne j$, so the $X_i$ must label distinct vertices in the frontier. Since any split separating $X_i$ and $X_{i+1}$ labels at least one edge in any frontier path between them, the number of edges in a minimal frontier path between $X_i$ and $X_{i+1}$ is at least the number of splits separating them. This then implies that the $X_i$ must be in order along the frontier, at the distances claimed. $\square$

**Fig. 8:**
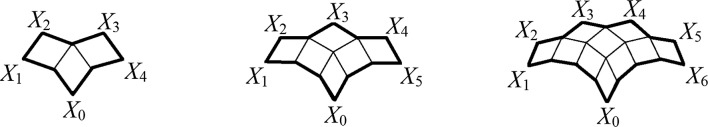
An *m*-dart, for $m=5,6,7$ respectively. The frontier edges, shown in bold outline, are characterized in the text. The outer vertices labelled by the $X_i$ are the corners. The point of the dart is the unique corner which is $m-3$ frontier edges away from the closest corners

Now suppose *C* is an *m*-cycle in $N^-$. If $m=4$, this lemma indicates that a frontier-minimal splits graph for the splits associated to *C* is also a 4-cycle, that is, the undirected version of the cycle. However, if $m\ge 5$, the splits graph is more complicated, having frontier as those depicted in the examples of Fig. 8. We refer to such blobs as *m*-*darts*. The *corners* of the *m*-dart are the vertices on the frontier of the dart that are labeled by sets of taxa $X_i$. The *point* of the *m*-dart, labelled by $X_0$, is the unique corner that is $m-3$ frontier edges away from its two closest corners. Thus in a closed walk around the frontier of the dart starting at the point, the number of edges between consecutive corners is

$$\begin{aligned} m-3,1,2,2,\dots ,2,2,1, m-3. \end{aligned}$$

Putting all this together, we have the following.

### **Theorem 34**

*Given a level-1 unrooted network *$N^-$,* the frontier of any frontier-minimal splits graph for *$S(N^-)$* is the graph obtained from *$N^-$* by the following steps:*

1. *Contract any 2- and 3-cycles,*
2. *Undirect the hybrid edges in any 4-cycles,*
3. *Replace any m*-*cycle, *$m\ge 5$,* with the frontier of an m*-*dart so that the point is at the hybrid node and with the m cut edges incident to the cycle connected to the corners of the dart in the same circular ordering as in the cycle.*

### *Proof*

By Lemma 25, we may assume $N^-$ has no 2- or 3-cycles. Let *k* denote the number of cycles of size $\ge 4$ on $N^-$, and *G* a frontier-minimal splits graph for $\mathcal {S}(N^-)$.

By Corollary 31, the tree of blobs of $N^-$ and the tree of blobs of *G* are isomorphic, so we identify them. Moreover, since cycles in $N^-$ are vertex-disjoint, each cycle of size $m\ge 4$ on $N^-$ gives rise to a node of degree *m* in the tree of blobs, so the tree of blobs has *k* multifurcations. This implies *G* has at least *k* blobs. *A priori* it is possible that *G* has more than *k* blobs, since if two blobs in *G* shared a vertex they would be collapsed to a single node in the tree of blobs.

By Proposition 30 property (3), frontier edges of G colored by splits associated with a single cycle of $N^-$ all lie in a single blob of *G*, since Lemma 32 shows two such cycle splits are either incompatible, or both incompatible with a third. Moreover, since the tree of blobs of $N^-$ (and *G*) has exactly *k* vertices corresponding to cycles in $N^-$, it follows that *G* has exactly *k* blobs, which are vertex disjoint, and each blob has only splits associated to a single cycle of $N^-$ coloring its frontier edges. This establishes a one-to-one correspondence between cycles in $N^-$ and blobs in *G*, according to the coloring of frontier edges

Fixing a cycle *C* on $N^-$, and contracting all edges of *G* not labeled by splits associated to *C* preserves the frontier of the blob of *G* corresponding to *C*. By Lemma 33, this frontier is either a 4-cycle (if $m=4$) or an *m*-dart (if $m\ge 5$). Moreover, the partition of *X* according to the connected components of $N^-$ with *C* deleted is the same as that from the labeled corners of the 4-cycle or *m*-dart, with the same circular ordering, and in the case $m\ge 5$ the descendants of the hybrid node of *C* label the dart’s point. Thus both *C* in $N^-$ and the blob of *G* associated to *C* must map to the same multifurcation in the tree of blobs, and the frontier of *G* must have the form described. $\square$

**Fig. 9:**
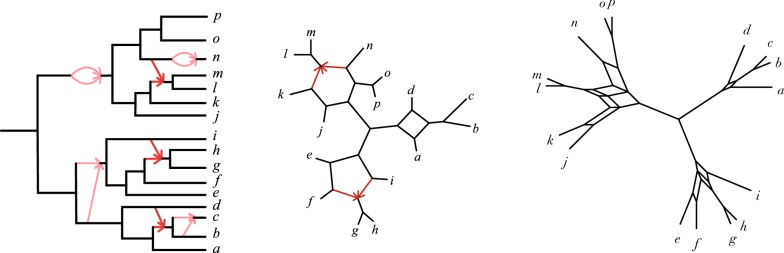
(L) A rooted level-1 network $N^+$ with 2- and 3-cycles shown in light red, (C) the unrooted topological network $N^-$ obtained from $N^+$ by contracting 2- and 3-cycles and undirecting 4-cycles, and (R) a frontier-minimal splits graph that corresponds to $N^-$ by Theorem 34. Note that the splits graph has a 4-cycle, a 5-dart, and a 6-dart, arising from the 4-, 5-, and 6-cycles of $N^-$. The metric structure of the splits graph, which is not described by Theorem 34, reflects the split weights as defined by Definition 18. See also Example 37

Figure 9 illustrates this theorem for a particular network. Note that the theorem only describes the topological structure of the splits graph. The metric splits graph’s structure depends on details of the network beyond the analysis of the theorem, as is seen in Definition 18 of the split weights.

Importantly for applications, one can apply Theorem 34 “in reverse” to obtain information about the network $N^-$ from the frontier-minimal splits graph for $\mathcal {S}(N^-)$. Indeed, although the correspondence between level-1 networks $N^-$ and frontier-minimal splits graphs as described in Theorem 34 is not one-to-one, the only information lost from $N^-$ is that of the existence of 2- and 3-cycles and the determination of the hybrid node in a 4-cycle. The specific geometry of the frontier of an *m*-dart in *G* for $m\ge 5$ allows one to identify such *m*-cycles and hybrid nodes in $N^-$. In conjunction with previous sections of this paper, this recovers the main result of [3]:

### **Corollary 35**

*Under the NMSC model on a level-1 network *$N^+$,* for generic parameters, the network obtained from *$N^-$* by suppressing 2- and 3-cycles and undirecting 4-cycles is identifiable.*

Beyond providing a different argument for this corollary, Theorem 34 provides theoretical underpinnings to a practical algorithm for (partial) network topology inference from a sample of gene trees, as outlined in the next section.

## The NANUQ algorithm for inference of phylogenetic networks

Here we revisit and formalize the NANUQ algorithm sketched in the introduction.

### **Algorithm**

*(NANUQ*) Input: A collection of unrooted topological gene trees on subsets of a taxon set *X*, such that each 4-element subset of *X* appears on at least one tree; and two hypothesis testing levels $0<\alpha ,\beta <1$.

1. For each subset of 4 taxa, determine the empirical quartet counts across the gene trees for each of the 3 resolved topologies. If all four taxa are not on a gene tree, that tree does not contribute to the counts. These 3 counts form an empirical *quartet count concordance factor* (qcCF) vector for the 4 taxa.
2. For each set of 4 taxa, apply two statistical hypothesis tests to its qcCF, with levels $\alpha ,\beta$, as described below, to determine whether to view the qcCF as supporting (1) a star tree, (2) a resolved tree, or (3) a 4-cycle network on the taxa. In cases (2) and (3), use the maximum likelihood estimate of the topology from the qcCF to determine which tree or network is supported.
3. Use the quartet networks/trees from the previous step to construct a network quartet distance between taxa, as in Definition 22, with the modification described below for unresolved quartets.
4. Use the NeighborNet Algorithm [6] to determine a weighted circular split system approximating the quartet distance.
5. Use the Circular Network Algorithm [7] to determine a frontier minimal splits graph for the circular system.

Output: A splits graph to interpret via Theorem 34 for features of $N^+$.

To analyze the running time for this algorithm, suppose $|X|=n$ and the input set contains *m* trees. First note that tallying displayed quartets in Step 1 can be done in time $\mathcal {O} (n^4m)$, as discussed in [5]. The hypothesis tests for Step 2 are performed in constant time for each set of 4 taxa, for a total of $\mathcal {O} (n^4)$. Step 3 in which the distance is computed requires running through the inferred quartet trees and networks for an additional time of $\mathcal {O} (n^4)$. For Step 4, the NeighborNet algorithm as presented in [6] takes time $\mathcal {O} (n^3)$. (The software implementation is different, having a guaranteed running time that is only exponential in *n*, but that in practice is much faster). Since NeighborNet can produce positive weights for all $\mathcal {O} (n^2)$ splits consistent with some circular ordering of the taxa, results from [7] show that the time for the Circular Network Algorithm in Step 5 is $\mathcal {O} (n^4)$. Thus the total time for NANUQ is $\mathcal {O} (n^4m)$.

We implemented Steps 1, 2, and 3 of the NANUQ algorithm in an R package MSCquartets, with a function accepting an input file of (metric or topological) Newick gene trees, and producing an output file of the network quartet distances computed from this data. When this file is opened by SplitsTree4 [8], Steps 4 and 5 are performed. With these implementations, we have found Step 1 by far dominates computational time, as is consistent with the running time analysis. However, the use of R probably slows computations considerably over what could be achieved.

The R package MSCQuartets is currently available on request from the authors, and has been submitted to CRAN for downloading.

### Testing empirical quartet counts

The statistical tests in Step 2 of the NANUQ algorithm, based on [4], require further explanation.

We use a hypothesis testing framework, in which two tests are performed. One test is used to decide whether the topological signal in a qcCF is strong enough to justify belief in any resolved network or tree, as opposed to viewing the quartet as unresolved. The second test is used to decide if the qcCF supports a 4-cycle network or a tree. The particular network or tree is then chosen via maximum likelihood.

These tests are performed for each set of four taxa, as if all quartet gene trees are independent. Of course, these are not independent, since the quartet trees are subtrees of the same gene trees, and under the NMSC these gene trees are assumed to have formed on the same species network. Since the lack of independence depends in part upon the species network parameter, which is unknown and sought, it is not clear how one might compensate for it. However, treating summary statistics as independent when they are not also underlies phylogenetic inference schemes built on *pseudo*-likelihood (e.g., SNaQ) and seems a necessary and acceptable concession for developing fast and tractable methods.

Suppose for a set of 4 taxa, one has tabulated the counts of the quartets displayed on gene trees in a sample, obtaining the qcCF. Under the NMSC model, these counts can be viewed as a multinomial sample from the distribution determined by the theoretical CF. Normalizing by the total count, we obtain an empirical CF which estimates the theoretical one. Because this empirical CF is computed from a finite sample, it is unlikely that it lies exactly where the theoretical CF would as shown in Fig. 4. However, an appropriate statistical test can be used for deciding whether the qcCF supports a quartet tree or network under the NMSC.

Specifically, for a fixed qcCF we first perform a hypothesis test for a star tree. More formally, under the NMSC the null hypothesis is

$$\begin{aligned} H_0\text{:}\text{ The qcCF arises from a 4-taxon star tree.} \end{aligned}$$

The alternative hypothesis is that the qcCF may have arisen from either a resolved tree or a network under the NMSC, or that the NMSC model somehow does not apply. The NANUQ algorithm focuses exclusively on the first interpretation of the alternative, assuming that all data arises from the NMSC.

As the star tree has theoretical CF (1/3, 1/3, 1/3), we perform this test by computing the likelihood ratio statistic from the three quartet counts in qcCF, using a $\chi ^2$ distribution with 2 degrees of freedom to compute a *p*-value. With level $\beta$ chosen for the test, we reject the star tree hypothesis for *p*-values smaller than $\beta$. (Note that $\beta$ is used here as the size of the rejection region for the test, *not* the probability of a type II error). For larger *p*-values, we fail to reject the star tree.

As will be shown in Theorem 36 below, under the NMSC on a binary level-1 network for any level $\beta > 0$, the probability that this test always rejects quartet star trees, approaches 1 as the sample size (number of gene trees) goes to infinity. Nonetheless, with finite and noisy data (perhaps due to gene tree inference error), this test is important to prevent interpreting a qcCF that is nearly uniform from indicating support for a particular tree or network topology. Performing this test allows for the suppression of weak and possibly erroneous signals in data sets of finite size.

The second hypothesis test is to assess support for a tree-like quartet vs. a 4-cycle. Under the NMSC, we formulate a null hypothesis of

$$\begin{aligned} H_0\text{:}\text{ The qcCF is tree-like}, \end{aligned}$$

with alternative that qcCF is not tree-like. Since underlying the NANUQ algorithm is the assumption that gene tree data arose from the NMSC, rejecting the null hypothesis is interpreted as giving evidence that the quartet network has a 4-cycle. That is, rejecting the null hypothesis is interpreted by NANUQ as support for a 4-cycle quartet network, ignoring the (measure 0) region where non-tree-like CFs from $3_2$-cycles may coincide with 4-cycle CFs.

Geometrically, the model for this null hypothesis is the 3 line segments in the simplex of Fig. 4(L), with the alternative model the complement of the 3 line segments as shown in Fig. 4(R). For the test, we compute the likelihood ratio statistic for these hypotheses. Using a $\chi ^2$ distribution with 1 degree of freedom (the asymptotic distribution for a resolved tree) would be a standard approach to obtain a *p*-value for the statistic. However, the model space for $H_0$ has a singularity at the center of the simplex, and justification for the $\chi ^2$ depends on the model being approximated well by its tangent line. As this approximation fails at the singularity, using a $\chi ^2$ approximation in the vicinity of the singularity may result in poor testing, which in this case is quite conservative. Although the neighborhood of the singularity on which the $\chi ^2$ behaves poorly shrinks as the sample size *m* grows, this ‘bad’ neighborhood is present for any finite sample size. However, this particular model and its special geometry at the singularity has been studied extensively in [4], where an alternative approximate distribution has been developed. We adopt the techniques of that work for use with the likelihood ratio statistic, to compute *p*-values.

For the NANUQ algorithm with level $\alpha$ for this test, we interpret a *p*-value greater than $\alpha$ as support for a tree, with the particular tree topology chosen as the maximum likelihood estimate from the qcCF. The MLE quartet tree topology is simply the quartet topology with the largest count in the qcCF. A *p*-value less than $\alpha$ is interpreted as support for a 4-cycle network, where the particular 4-cycle topology supported is the maximum likelihood estimate from the qcCF. This is determined by which of the 3 triangular regions in the simplex the normalized qcCF lies, as in Fig. 4(R).

With two tests being performed in this way, it is possible that for a particular set of 4 taxa we find that we fail to reject the first hypothesis (that the qcCF arises a star tree) but reject the second (that it arises from a tree). This can be forced to occur by taking $\beta$ quite small while $\alpha$ is large, but it may occur for less extreme values. In such a situation one must give priority to one test over the other. We choose to prioritize the first test, so that in this case we view the tests as supporting a star tree, on the principle that evidence for hybridization should be judged by the strictest standards.

The output of NANUQ depends on the choices of significance levels $\alpha$ and $\beta$, with smaller values of $\alpha$ requiring stronger evidence for 4-cycles, and smaller values of $\beta$ requiring stronger evidence for any resolution of the 4-taxon network. We view this feature positively, as it requires that users of NANUQ examine their data and consider the impact of choosing different levels. Since the input gene trees are likely to be noisy from the error introduced by inferring them from gene sequences, it is reasonable to set $\alpha$ quite small, which imposes a high standard for evidence of hybridization. However, practitioners must decide (and report) what standards they impose by their choices of $\alpha$ and $\beta$.

We note also that there is no reason that $\alpha$ and $\beta$ should be chosen to have equal values, and we believe appropriate choices of both will depend upon the level of noise in the data. In particular, *a priori* choices of conventional values such as 0.05 are likely poor choices. Investigating the impact of a range of choices for $\alpha$ and $\beta$ on the final splits graph is a necessary part of the analysis. This issue is addressed briefly below through several examples of simulated and empirical data sets, but we defer more complete comments to a future paper directed at empiricists.

The testing framework described here treats any qcCF judged non-tree-like as supporting a 4-cycle and not a $3_2$-cycle. Using Proposition 10, by an assumption of sufficiently long edges descended from all hybrid nodes, one can rule out the possibility of non-tree-like $3_2$-cycles, although an empiricist may prefer not to make such an assumption. In a future version of NANUQ we intend to offer a choice of using an additional statistical test for $3_2$-cycle networks, but this test will also be nonstandard, due to the model having a singularity at the crossing of three line segments (see Fig. 4(C)), and thus requires additional theoretical development.

Finally, we note that these tests take into account the total number of quartets for a particular set of four taxa. If some gene trees have missing taxa, these numbers may vary with the set of four taxa, but the tests can still be performed. Thus such missing taxa will not be problematic for performing the algorithm, and moderate levels of missingness should not greatly degrade performance.

### Quartet distance with unresolved quartets

The quartet distance defined for a binary network earlier in this work required that all quartet networks, after contraction of 2- and 3-cycles, be binary, with positive lengths for all tree edges. However, in Step 2 of the NANUQ algorithm we include a hypothesis test for a star tree, to reduce the possibility of supporting a particular resolved tree or 4-cycle when the qcCF is nearly uniform and gives at best weak evidence as to what the resolved topology should be. Additionally, one might sample multiple individuals per taxon, which can be thought of as polytomies at the leaves of the species network. (See, for instance, Example 39). We thus must explain how we modify the quartet distance computed in Step 3 to handle unresolved quartets.

To this end, we make a simple extension of Definition 20 for $\rho _{xy}(Q_{xyzw})$. Guided by the results in [5] on quartet distances for non-binary trees, we set

$$\begin{aligned} \rho _{xy}(Q_{xyzw})=1 \text{ if }{\widetilde{Q}}_{xyzw}\text{ is a star tree.} \end{aligned}$$

In particular, this means a star tree is viewed as separating any two distinct taxa on it.

Under the assumption of a binary network, this modification has no impact on the asymptotic behavior of the algorithm under the NMSC model, since by Theorem 36 below the probability of rejecting all quartet star trees approaches 1 as the size of the data set grows.

### Statistical consistency

An estimator of a model parameter is said to be statistically consistent if the probability of inferring the parameter to arbitrarily small precision from a data set of size *m* produced in accord with the model approaches 1 as *m* approaches infinity. Since the NANUQ algorithm depends upon choices of two significance levels, $\alpha$ and $\beta$, these choices must be taken into account in formulating an appropriate notion of consistency for it. As we will show, because of the assumption that the unknown network is binary, the value of $0<\beta <1$ will be inconsequential for this notion, since as *m* grows the probability of rejecting a quartet star tree approaches 1 for every choice of four taxa.

In contrast, when a true quartet network is tree-like, then no matter how large the data set, we expect to reject the null hypothesis that the corresponding qcCF is tree-like approximately 100$\alpha$% of the time. That is, with probability about $\alpha$, the hypothesis test will incorrectly support a 4-cycle network when the true quartet network is tree-like. This behavior is fundamental to the hypothesis testing framework, and cannot be avoided.

As a consequence, any notion of statistical consistency for NANUQ must consider sequences of significance levels $\alpha _m \rightarrow 0$. We will show the existence of a sequence of levels $\alpha _m$, dependent on the sample size *m*, so that as *m* increases the probability of correctly failing to reject the null hypothesis (avoiding type I errors at level $\alpha _m$) approaches 1 while at the same time the probability of correctly rejecting the null hypothesis (avoiding type II errors) also goes to 1. The following theorem then captures the sense in which NANUQ is statistical consistent.

#### **Theorem 36**

*Under the NMSC model on a binary level-1 metric phylogenetic network* $N^+$,* for numerical parameters in which all induced quartet networks with *$3_2$-*cycles are tree-like, there exists a sequence* $\alpha _1,\alpha _2,\dots$,* with *$0<\alpha _m<1$ *and *$\alpha _m\rightarrow 0$ *such that for any *$0<\beta <1$* the NANUQ algorithm with significance levels *$\alpha _m$* and *$\beta$* on a data set of m gene trees will, with probability approaching 1 as *$m\rightarrow \infty$,* infer the binary unrooted phylogenetic network associated to *$N^+$* by Theorem*  34.

#### *Proof*

It is enough to show that the $\alpha _m$ can be chosen so that with probability approaching 1 the quartet distance computed in the NANUQ algorithm exactly agrees with the theoretical quartet distance for the true network $N^+$. As suggested above, this will follow from showing that as the sample size $m \rightarrow \infty$ with probability approaching 1, the hypothesis tests performed will (1) reject a star tree at level $\beta$, and (2) fail to reject a tree-like quartet network when the true one is tree-like, and reject a tree-like quartet network when the true one is non-tree-like at level $\alpha _m$.

Consider first the hypothesis test for a star tree for a particular choice of 4 taxa. The result we need is essentially a standard one, but we give a full argument as an orientation for the argument for the second test. Since the network is binary, the true multinomial parameter values are $CF = (p_1, \, p_2, \, p_3)$ with $p_i \ne 1/3$ or 0, and the null hypothesis is $H_0\text{:}\ CF = (1/3,\, 1/3,\,1/3)$. The test statistic is $\lambda = -2 (\ell _0 - \ell )$ where $\ell _0$ is the supremum of the log-likelihood over parameter values in the null space [here only (1/3, 1/3, 1/3)], and $\ell$ is the supremum of the log-likelihood over the full simplex. The statistic $\lambda$ is asymptotically $\chi ^2$-distributed with 2 degrees of freedom.

A qcCF $(m_1,m_2,m_3)$ for a sample of size *m* is a multinomial sample from a distribution with parameters $(p_1, p_2, p_3)$. Then

$$\begin{aligned} \lambda&=2 \Big (m_1\log \left( \frac{m_1}{m}\right) +m_2\log \left( \frac{m_2}{m}\right) \\&\qquad +m_3\log \left( \frac{m_3}{m}\right) -m\log \left( \frac{1}{3}\right) \Big )\\&=m\cdot 2 \Big (\frac{m_1}{m}\log \left( \frac{m_1}{m}\right) +\frac{m_2}{m}\log \left( \frac{m_2}{m}\right) \\&\qquad +\frac{m_3}{m}\log \left( \frac{m_3}{m}\right) -\log \left( \frac{1}{3}\right) \Big )\\&=m X_m, \end{aligned}$$

where $X_m$ is a random variable. By the law of large numbers and the continuous mapping theorem $X_m$ converges in probability to

$$\begin{aligned} c=2\, \big (p_1\log p_1+ p_2\log p_2+ p_3\log p_3 -\log (1/3) \big )>0. \end{aligned}$$

Thus for any $\epsilon >0$ there exits an *M* such that $m>M$ implies ${\mathbb {P}}(X_m>c/2)>1-\epsilon$, and consequently, that ${\mathbb {P}}(\lambda>mc/2)>1-\epsilon .$ This means that for any significance level $0<\beta <1$, the null hypothesis will be rejected for *m* sufficiently large with probability at least $1 - \epsilon$. Since $\epsilon$ was arbitrary, as $m\rightarrow \infty$ the probability of rejecting the null hypothesis goes to 1. Since there are only finitely many 4-taxon subsets, the probability of rejecting that any of these are star-like also goes to 1.

Turning now to the hypothesis test for a tree-like quartet network on 4 specific taxa, suppose first the true CF is tree-like. The likelihood ratio statistic is judged using the approximating distribution (dependent on the sample size *m*) of the random variable $W_m=W$ described in Theorem 3.1 of [4]. Since the true network is binary, from results in that paper $W_m$ has a limiting distribution as $m\rightarrow \infty$, which is $\chi ^2_1$. To ensure that the probability of failing to reject the null hypothesis approaches 1 as $m\rightarrow \infty$, it is enough to choose any sequence of significance levels with $\alpha _m\rightarrow 0$.

In contrast, if the true CF is non-tree-like, we must pick significance levels more carefully. Without loss of generality, suppose the true CF is $(p_1,p_2,p_3)$ with $p_1\ge p_2>p_3$. A qcCF $(m_1,m_2,m_3)$ for a sample of size *m*, with $m_{\max }=\max (m_i)$, yields a likelihood ratio statistic

$$\begin{aligned} \lambda&=2 \, \Big (m_1\log \left( \frac{m_1}{m}\right) +m_2\log \left( \frac{m_2}{m}\right) \\&\qquad +m_3\log \left( \frac{m_3}{m}\right) - m_{\max }\log \left( \frac{m_{\max }}{m}\right) \\&\qquad -(m-m_{\max })\log \left( \frac{m-m_{\max }}{2m}\right) \Big )\\&=m\cdot 2 \, \Big (\frac{m_1}{m}\log \left( \frac{m_1}{m}\right) +\frac{m_2}{m}\log \left( \frac{m_2}{m}\right) \\&\qquad +\frac{m_3}{m}\log \left( \frac{m_3}{m}\right) - \frac{m_{\max }}{m}\log \left( \frac{m_{\max }}{m}\right) \\&\qquad -\left( \frac{m-m_{\max }}{m}\right) \log \left( \frac{m-m_{\max }}{2m}\right) \Big )\\&=m \, Y_m. \end{aligned}$$

where $Y_m$ is a random variable. But $Y_m$ converges in probability to

$$\begin{aligned} d=2( p_2\log p_2 +p_3\log p_3 -(p_2+p_3)\log ((p_2+p_3)/2) ) >0. \end{aligned}$$

Thus for any $\epsilon >0$ there exits an *M* such that $m>M$ implies ${\mathbb {P}}(Y_m>d/2)>1-\epsilon$, and thus that ${\mathbb {P}}(\lambda>md/2)>1-\epsilon .$ Let $\alpha ^{\prime}_{m} ={\mathbb {P}}(W_m>md/2)$. Then we have that for any $\epsilon >0$ there exists an *M* such that for $m>M$ the probability of rejecting the null hypothesis at level $\alpha ^{\prime}_{m}$ is $>1-\epsilon$. Thus as $m\rightarrow \infty$ the probability of rejecting the null hypothesis goes to 1. As the $W_m$ converge in distribution to a $\chi ^2_1$, one also sees that $\alpha ^{\prime}_{m} \rightarrow 0$.

Since there are a finite number of non-tree-like subsets of 4 taxa, we choose $\alpha _m$ to be the minimum of the $\alpha ^{\prime}_{m}$ for these subsets, to ensure the probability of rejecting the null hypothesis for all of them goes to 1 as $m \rightarrow \infty$. As $\alpha _m\rightarrow 0$, this sequence has all the desired properties. $\square$

Note that the assumption in the theorem that all $3_2$-cyles are tree-like can be ensured through, for example, Proposition 10, by requiring that no edges descending from hybrid nodes have length less than $\log (5/4)$.

Although we do not give a formal proof here, NANUQ remains statistically consistent even in the absence of incomplete lineage sorting. Informally, one can “turn off” ILS in the multispecies coalescent model by shrinking all population sizes on the species network. Equivalently, if the species network’s branch lengths, measured in coalescent units, go to $\infty$, then the distribution of rooted topological gene trees approaches that of a hybridization model with no ILS. One can thus establish consistency either by taking appropriate limits in the argument above, or by analyzing quartet concordance factors for the pure hybridization model directly.

### Variants of NANUQ

The NANUQ algorithm can be adapted to use any means of determining from data what 4-taxon species network is supported. Thus future developments might allow for the replacement of Steps 1 and 2 by alternative approaches. For instance, one might adopt for analyses of 4-taxon networks an invariants-based approach such as in [25], so that the data becomes aligned genomic sequences. Alternatively, generalizations of ideas from [26] which are now being investigated may allow for determination of rooted triple networks from genomic sequences, and a rooted triple distance can replace the quartet distance used here. The essence of the NANUQ approach is to use a quartet (or rooted triple) distance appropriate to networks along with the NeighborNet and Circular Network Algorithm, though how one obtains the information necessary to compute the distance may vary.

### Sources of error

While NANUQ is a statistically consistent (in the precise sense of Theorem 36) method of inferring certain network features from a collection of gene trees produced by the NMSC model, in practice it must be applied to a finite set of inferred gene trees. Possible sources of errors in conclusions drawn from NANUQ include:

1. Error in gene trees, due to their inference from sequence data,
2. Small sample size (e.g., few gene trees, many missing taxa on gene trees),
3. Miscalls of evidence for/against hybridization in individual quartets, in Step 2,
4. The NeighborNet Algorithm’s projection of the split system onto a circular one, in Step 4,
5. The presence of non-tree-like $3_2$-cycles on some induced quartet networks,
6. NMSC model misspecification due to any of:
  - A non-level-1 network,
  - Structure within populations,
  - Continuous gene flow between populations.

Thus one should not expect empirical data to necessarily lead to a splits graph exactly conforming to form described by Theorem 34.

Note that the algorithm of [27] offers an alternative to NeighborNet that might reduce the error arising in passing to a circular split system from the quartet distance. However, this has not been implemented in general purpose software yet, so we were unable to test its performance.

We have chosen not to suggest any automatic interpretation of the output of NANUQ, such as a mechanism for producing the closest splits graph (by some measure) that conforms exactly to the form described by Theorem 34. Thus the user must visually consider the output, which will reflect some of the error. In particular, SplitsTree offers a capability of removing splits with small weight from a splits graph, and this can be useful for removing some of the noise remaining after projecting onto a circular split system.

## Examples

In this section we present three examples of data analysis with NANUQ. The first uses a simulated data set of gene trees (without any gene tree inference error), the second the well-known and well-studied yeast data set of [28], and the third the butterfly data set of [29]. For the empirical data sets, we use gene trees previously inferred from genetic sequences. Reported running times are from a Macbook Pro computer with a 3.1 GHz processor.

**Table 1:** The metric species network $N^+$, in extended Newick format, used for simulating gene trees under the NMSC model

(((((a:1.5,(((b:.8,h1#.5:.1)x1:.2,(c:.7)h1#.5:.3)x2:.3)h2#.5:.2)x3:1.5,(h2#.5:.2,d:1.5)x4:1.5)x5:2,h3#.5:1.5)x6:0.5, (((e:2,(f:1,((g:.25,h:.25)x7:.25)h4#.5:.5)x8:1)x9:1,(h4#.5:.5,i:1)x10:2)x11:0.5)h3#.5:2)x12:1,((((j:4.5,(k:3.5,((l:2.75,m:2.75)x13:.25) h5#.3:.5)x14:1)x15:1,((((n:1)h6#.5:2,h6#.5:2)x16:.5,h5#.3:.5)x17:1,(o:3.5,p:3.5)x18:1)x19:1)x20:.25)h7#.5:.5,h7#.5:.5)x21:.25)r	

The topology of $N^+$ is shown in Fig. 9(L)

### *Example 37*

We generated a data set of 1000 gene trees using Hybrid-Lambda [30] on the species network $N^+$ shown in Fig. 9(L), with branch lengths in coalescent units and hybridization parameters as shown in Table 1.

**Fig. 10:**
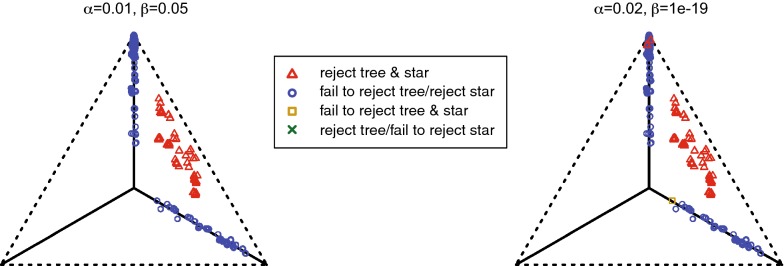
Representative simplex plots for empirical CFs, with hypothesis testing results, computed from a simulated data set of 1000 gene trees from the species network given in Table 1

In running NANUQ on this data set, our implementation of Steps 1-3 in R required about 63*s* of computation time. We considered a range of values of $\alpha$ and $\beta$ for the hypothesis tests. To visualize outcomes of the hypothesis tests, we produced simplex plots such as those shown in Fig. 10, which plot empirical CFs (i.e., qcCFs normalized to sum to 1) for each set of 4 taxa, color coded to indicate test outcomes. The results of the hypothesis tests gave a rather clean separation of empirical CFs into those close to the 3 line segments which were classified as tree-like, and those farther away which were viewed as supporting a 4-cycle. We found that for any level $\alpha$ in the range $10^{-17}\le \alpha \le .01$, our hypothesis tests drew the same conclusions as to which qcCFs supported a 4-cycle (red triangles). The close clustering of the qcCFs not rejected as tree-like (blue circles) around the tree model also suggests little error in them, so that a rather large value of $\beta$ might be sufficient to test for lack of resolution. When $\beta$ is set to .05, all qcCFs result in rejection of the star tree hypothesis. As shown in the figure, when $\beta$ is reduced to $10^{-19}$ a single failure to reject the star tree hypothesis occurs (tan square). Using $\alpha =.01$ and $\beta =.05$ to compute the quartet distance, from SplitsTree4 we obtain the splits graph shown earlier as Fig. 9(R). Under the rules of Theorem 34, this correctly gives all features of $N^-$ inferable by NANUQ, as shown in Fig. 9(C).

Reducing the sample size to 300 gene trees, while using the same values of $\alpha$ and $\beta$, we obtained the same correct inference result.

### *Example 38*

For the second example we use gene trees inferred from a subset of the yeast data set of [28] which have been analyzed by multiple investigators [9, 10, 31–34]. The 106 gene trees each relate a single allele sampled from seven *Saccharomyces* species: *S. cerevisiae* (S cer), *S. paradoxus* (S par), *S. mikatae* (S mik), *S. kudriavzevii* (S kud), *S. bayanus* (S bay), *S. castellii* (S cas), *S. kluyveri* (S klu), and the outgroup fungus *Candida albicans* (C alb). Running time for NANUQ’s Steps 1–3 was under 0.5 s.

**Fig. 11:**
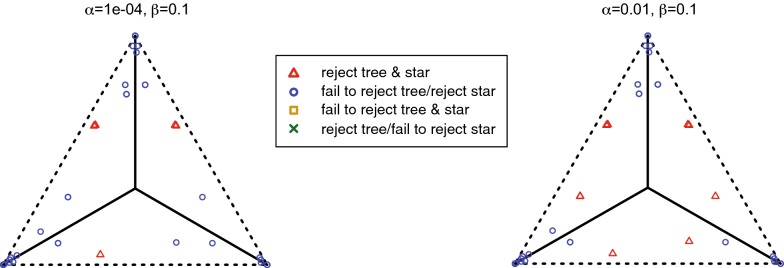
Simplex plots for hypothesis test results on the yeast data set, with two choices of significance levels $\alpha =10^{-4}$ and $10^{-2}$ with $\beta =0.1$. The choice of $\beta$ here is largely irrelevant, as no plotted empirical CFs are near the center. Larger $\alpha$ results in more empirical CFs being determined as supporting 4-cycles, as several blue circles on the left change to red triangles on the right

Displayed in Fig. 11 are some sample results from hypothesis tests for several choices of $\alpha$ and $\beta$. As all of the empirical CFs are far from $(1/3, \, 1/3, \, 1/3)$, the CF for the star tree, only a quite small $\beta$ would lead to failing to reject the star tree for any set of 4 taxa. Thus, for this data set, we set $\beta =0.1$ and classify all quartet networks as resolved, either as trees or 4-cycle networks. (We also see that no empirical CFs are plotted near the locations of non-tree-like $3_2$-cycle CFs, giving us some confidence in NANUQ’s assumption that there are none in the data). We chose values of $\alpha =10^{-4}$ and $10^{-2}$ as the first of these results in only the most extreme empirical CFs (far from the tree-like CF line segments) being interpreted as supporting 4-cycle networks, while the larger value, in imposing a less strict standard for evidence of hybridization, classifies more of those empirical CFs distant from the null model as 4-cycles. Further increasing $\alpha$ to values $>.08$ would result in additional classification of 4-cycle networks, but we chose to interpret those deviations from tree-like-ness as due to stochastic (or other) noise.

**Fig. 12:**
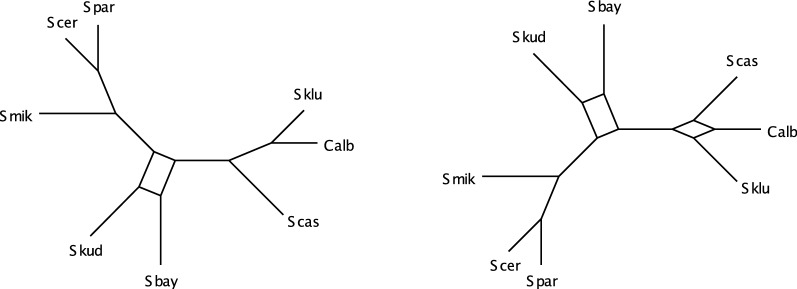
Networks inferred by NANUQ for yeast data of Example 38 with $\beta =0.1$ and $\alpha =10^{-4}$ (L) or $10^{-2}$ (R)

For each of the choices of $\alpha$, $\beta$, the splits graphs produced in NANUQ’s use of SplitsTree are shown in Fig. 12. Since these show only 4-cycles, they can be directly interpreted as indicating the undirected version of the true level-1 network topology relating the taxa, with all 2- and 3-cycles contracted. We obtain no information on root location from NANUQ since no cycles have size larger than 4.

### *Example 39*

For the third example we use gene trees inferred from a *Heliconius* butterfly data set [29], also analyzed in [25], which have been presented as evidence of gene flow between sympatric species. The sequence data consists of 2909 loci, derived from non-overlapping 100-kb windows in the full genome of individuals. Four individuals were sampled from three ingroup *Heliconius* species: *H. rosina*, *H. melpomene*, *H. cydno* (labelled chioneus), and one individual from four outgroup species *H. ethilia*, *H. hecale*, *H. p. sergestus*, and *H. pardalinus*.

**Fig. 13:**
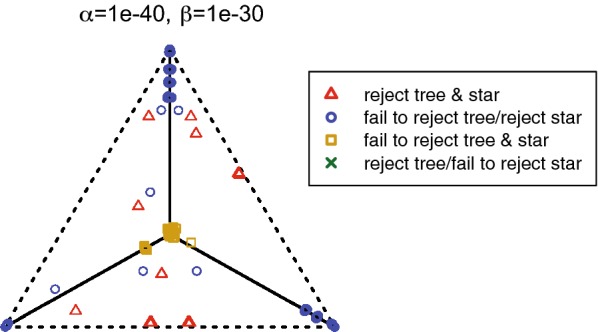
Simplex plot showing hypothesis test results for the *Heliconius* data set of Example 39

**Fig. 14:**
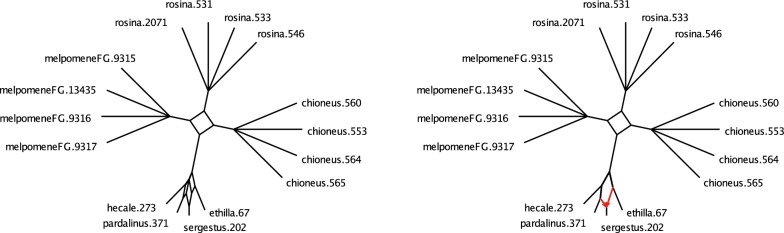
(L) Splits graph for *Heliconius* data set of Example 39, for $\alpha =10^{-40}$, $\beta =10^{-{30}}$, and (R) NANUQ inferred network structure

Running time for Steps 1–3 of the algorithm was about 174*s*. Figure 13 shows results of hypothesis tests for one choice of $\alpha$ and $\beta$, with Fig. 14 the resulting splits graph and inferred network structure. Note that a number of the empirical CFs (tan squares in Fig. 13) are close to the star-tree CF, and the choice of test level $\beta = 10^{-30}$ results in these being treated as unresolved quartets, giving the multifurcations in the splits graph for the three multi-sampled taxa. If $\beta$ is made larger so that star trees are rejected more often, then blobs can appear within the single taxon groups. For a broad range of choices for $\alpha$ and $\beta$ (not shown), the three ingroups *H. rosina*, *H. melpomene*, *H. c. chioneus* and the outgroup are related by a 4-cycle by NANUQ.

Though not the taxa of focus in the study [29], the splits graph of Fig. 14 depicts interesting relationships between the outgroup taxa and illustrates the flexibility of our analysis. While difficult to see in the SplitsTree4 output, there is a split with very small weight separating *H. ethilia*, *H. sergestus*, and *H. pardalinus* from the rest of the taxa. SplitsTree4 allows such small weight splits to be filtered out, and doing so leaves a 5-dart pointed at *H. sergestus*. However, for different values of $\alpha$ the 5-dart can change: for example, for $\alpha =10^{-17}$ the 5-dart points to *H. ethilla* instead. Thus while the central 4-cycle is very well supported, across many values of $\alpha$ and $\beta$, one might not want to draw firm conclusions on other hybridizations in this data set. The analysis does, however, suggest that the relationships between these taxa might warrant further investigation.

## Conclusions

The NANUQ algorithm is built on a broad collection of ideas, including theoretical understanding of the behavior of the network multispecies coalescent model on 4-taxon networks, hypothesis testing for certain submodels of the trinomial, a quartet-based intertaxon distance, circular split systems, and splits graphs. It provides a means of visualizing discordance in a collection of gene trees through a simplex plot, as well as fit to the coalescent on a level-1 species network in its splits graph output.

Importantly, NANUQ offers model-based, statistically consistent inference of most topological features of such a species network. Moreover, simulations and empirical examples suggest it is capable of performing network inference from gene trees rapidly in comparison to other approaches.

## Abbreviations

CF: concordance factor
ILS: incomplete lineage sorting
LSA: lowest stable ancestor
MSC: multispecies coalescent model
NANUQ: Network inference Algorithm via NeighbourNet Using Quartet distance
NMSC: network multispecies coalescent model
qcCF: quartet count concordance factor

## Footnotes

1 The word for “polar bear” in Iñupiaq and other Inuit languages, pronounced and sometimes written as ‘Nanook’.
